# Intelligent Photodetectors: Postmanufacturing Tunability toward Enhanced Performance and Advanced Functions

**DOI:** 10.1021/acs.chemrev.4c00763

**Published:** 2025-07-21

**Authors:** Yuanzhe Li, Xie He, Shih-Chi Chen, Ni Zhao

**Affiliations:** † Department of Electronic Engineering, 26451 The Chinese University of Hong Kong, Shatin, New Territories, Hong Kong SAR 999077, China; ‡ Department of Mechanical and Automation Engineering, The Chinese University of Hong Kong, Shatin, New Territories, Hong Kong SAR 999077, China

## Abstract

Photodetectors have become essential in modern technologies, yet traditional designs face challenges in many emerging applications owing to their fixed response characteristics, such as fixed responsivity, bandwidth, dynamic range, and spectral response. This limitation reduces their effectiveness when the target signal or ambient light varies in intensity, frequency, and wavelength, etc. Intelligent photodetectors, featuring postmanufacturing tunability, have emerged as a promising solution by allowing for real-time adaptability. These dynamic devices can adjust key performance metrics during operation, enhancing their versatility and applicability. Beyond basic photodetection, this adaptability lays fundamentals for various new functions, for instance, in-sensor computing and computational spectrum reconstruction. Some devices also offer switchable operation modes and integrated multisensory perception, minimizing the need for auxiliary components and simplifying the system architecture. This review aims to provide a comprehensive and holistic view of the diverse advances in intelligent photodetectors under the concept of tunable mechanisms. Beginning with foundational principles, we explore how postmanufacturing tunability enhances basic photodetection performance and enables advanced functions through tunable temporal response dynamics, spectral response, and multiple operational modes. We conclude with challenges and future opportunities, aiming to inspire continued innovation by bridging diverse fields of photodetection through the lens of postmanufacturing tunability.

## Graphical abstract

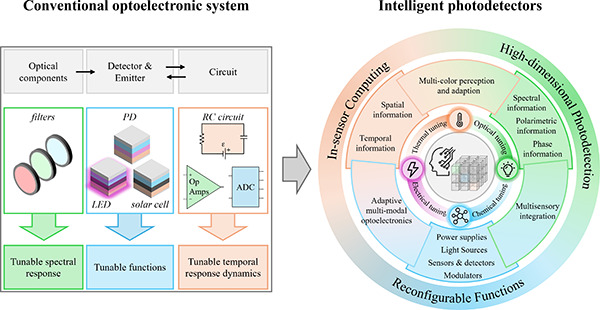

## 1 Introduction

In the field of optoelectronics, photodetectors are essential for converting light into electrical signals, making them critical components in applications like imaging,^1–8^ chemical analysis,^9–11^ and optical communications.^12–17^ However, traditional photodetectors are limited by their fixed response characteristics, which confines their effectiveness to specific, predefined conditions.^18–20^ This rigidity becomes a significant drawback in dynamic environments where light signals vary in intensity, wavelength, and other parameters (Figure 1a). For instance, a photodetector utilizing a carrier recycling mechanism for high photoconductive gain (and thus high responsivity) may struggle with high-frequency signals due to the extended carrier recombination time,^21–24^ and a device with fixed spectral response may be inadequate in scenarios like color imaging^25–30^ or even spectrum sensing.^31–40^ Additionally, the need for auxiliary components to perform tasks beyond basic photodetection complicates system design and integration.^41–43^ For instance, in an optical communication system, a photodetector cannot transmit feedback information without assistance from a light-emitting diode (LED). As a result, dual sets of LEDs and photodetectors are required to facilitate bidirectional communication, adding complexity to the system.^44^

**Figure 1:**
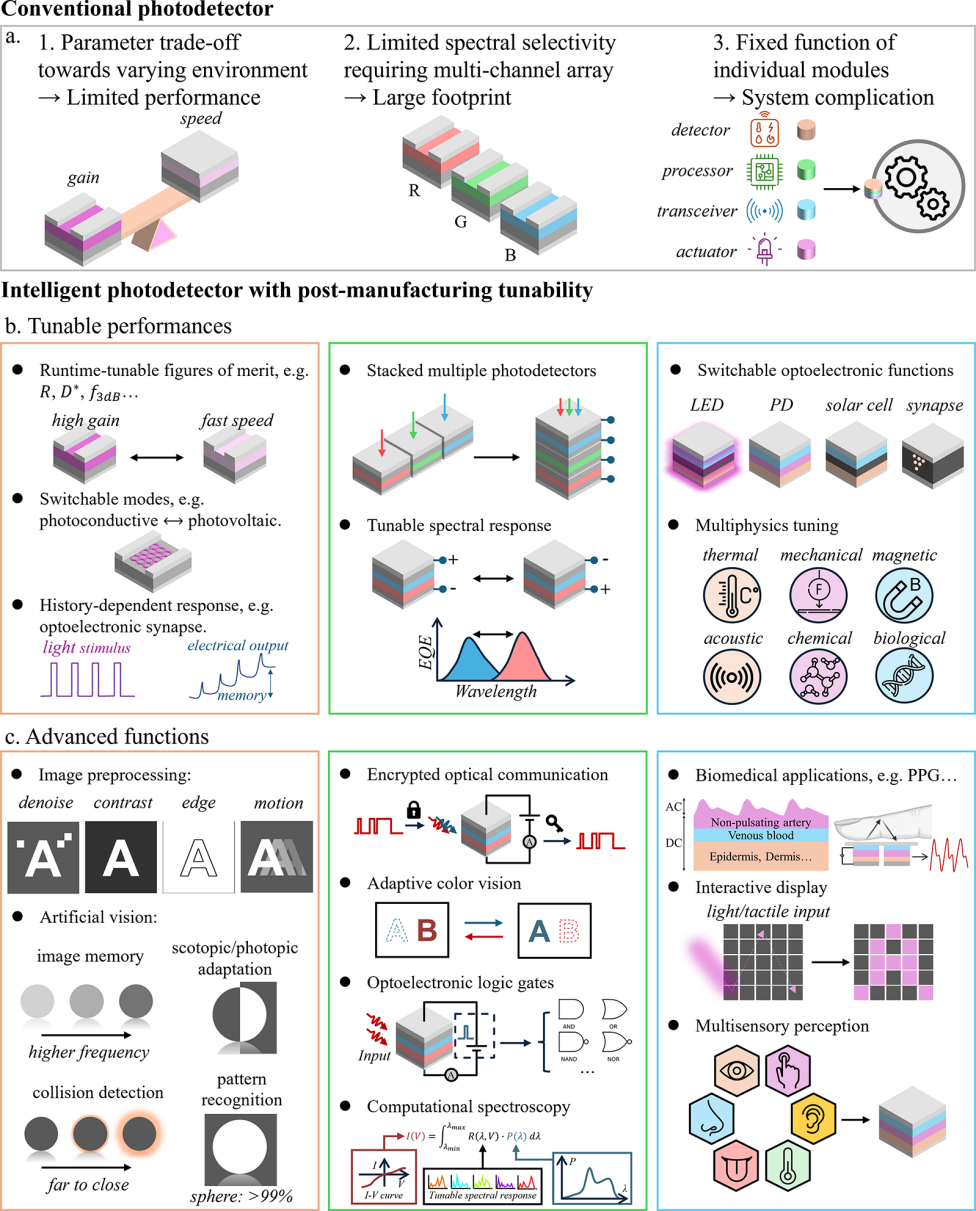
Comparison between conventional and intelligent photodetectors with postmanufacturing tunability. (a) Conventional photodetectors with fixed photoresponse relations and functions. (b) Tunable performances of intelligent photodetectors enabled by postmanufacturing tunability. (c) Advanced functions of intelligent photodetectors beyond basic photodetection enabled by postmanufacturing tunability.

Intelligent photodetectors offer a promising solution to these challenges, characterized by their postmanufacturing tunability. Unlike static photodetectors, whose response and spectral performance are fixed during fabrication through material engineering and structural design, intelligent photodetectors retain their flexibility to adjust performance in real-time during operation (Figure 1b). For detecting light of specific wavelengths, this adaptability includes not only the tuning of key metrics such as responsivity and speed but also switchable operation modes (e.g., photovoltaic and photoconductive),^45–48^ which can exhibit different responses to light (e.g., logarithmic or linear), enabling a broader range of modulation. By integrating memory mechanisms, intelligent photodetectors can further exhibit history-dependent characteristics,^49,50^ allowing their performance to be influenced by both the current conditions and previous experiences, facilitating more complex adaptability strategies. For those applications requiring multiple sets of spectral response, postmanufacturing tunability can be achieved by selectively reading out signals from stacked narrowband photodetectors^25–29^ or by operating a single photodetector under different bias voltages and/or polarities.^51–53^ The integration of light emitting, energy harvesting, neuromorphic sensing and multisensory perception functions in a single device enables multimodal operation and, to certain extent, minimizes the need of auxiliary components to perform complex tasks.^54–57^

Performance tunability of this new class of photodetectors not only enhances the photodetection performance but also opens up avenues for many advanced applications (Figure 1c). For example, runtime-tunable temporal response dynamics endows devices with analog computing capabilities, which are typically handled by subsequent circuits. This allows low-level image processing tasks—such as contrast enhancement,^58–60^ denoising,^61,62^ edge extraction,^63–66^ and motion detection^67–71^ —to be performed directly within the sensors. Devices with history-dependent response capabilities can mimic the functionality of human eyes, enabling high-level image processing such as memorization,^72–75^ vision adaptation,^76–81^ and pattern recognition.^82–92^ Similarly, tunable spectral response can eliminate the need for traditional optical components such as filters, facilitating broad applications, including encrypted optical communications,^14,93–97^ color perception,^39,98–100^ optoelectronic logic gates,^101–103^ and computational spectroscopy.^31–40,104^ Additionally, intelligent photodetectors with switchable functions extend their use beyond simple photodetection, enabling new applications such as bidirectional optical communication,^13,105^ vital sign monitoring,^13,106–115^ and interactive displays.^116^ By integrating various sensing technologies (e.g., optical, acoustic, tactile), they can function as multisensory sensors, offering a comprehensive understanding of the environment or subject being monitored.^16,55,117–129^ The above advancements effectively integrate functions previously handled by front-end optical components, separate detectors or emitters, and back-end circuits directly into the photodetector itself (Figure 2). When combined with time-division multiplexing strategies enabled by postmanufacturing tunability, new systems built on intelligent photodetectors can realize these complex functions with significantly reduced footprint, energy consumption, and fabrication complexity. The capability to function as multiple devices also enables potential multitasking, which greatly increases the system throughput and efficiency.

**Figure 2:**
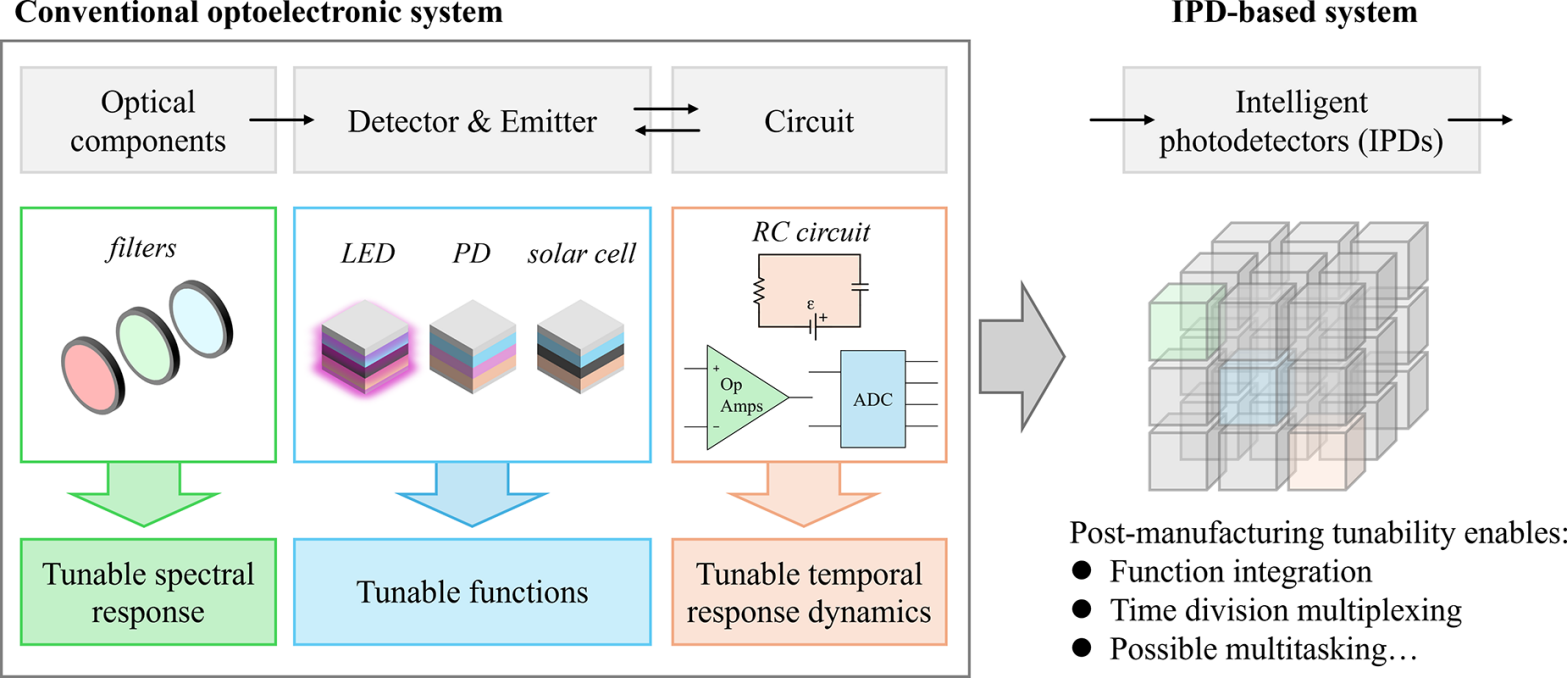
Evolution from a conventional optoelectronic system to an intelligent photodetector-based system. Function integration of frontend optical components, separate detectors or emitters, and backend circuits into a single device can be realized utilizing tunable spectral response, tunable functions, and tunable temporal response dynamics. The postmanufacturing tunability also brings the advantages of time division multiplexing and possible multitasking.

In this Review, we examine the principles and applications of intelligent photodetectors with postmanufacturing tunability. Our discussion emphasizes the shared features across various intelligent photodetectors and demonstrates how tunable performance metrics transform traditional limitations into opportunities for innovation. Section 2 begins with an overview of the device physics underlying conventional photodetectors and other common optoelectronics, providing the foundation for identifying tunable metrics that can drive the development of new intelligent photodetectors. In Section 3, we review the enhanced photodetection capabilities made possible by postmanufacturing tunability, showing how these adaptive devices surpass their static counterparts. Sections 4, 5, and 6 categorize current research on intelligent photodetectors based on the specific performance metrics they modulate during operation, highlighting how tunable performance can incorporate additional functions into photodetectors for advanced applications. Lastly, in Section 7, we summarize intelligent photodetectors by their degree of dynamism and conclude with insights into the remaining challenges and future directions.

## 2 Overview of Intelligent Photodetectors

The defining feature of intelligent photodetectors is their postmanufacturing tunability, which enables dynamic adjustment of performance metrics at runtime. This tunability is determined by both device design and the operation scheme.

In general, intelligent photodetectors are built upon or derived from classic photodetector structures such as photoconductors, photodiodes, phototransistors, and photovoltage FETs, as illustrated in Figure 3a. Their tunability involves standardized figures of merit (responsivity, bandwidth, dynamic range, etc.),^24,130^ as well as specialized parameters of specific detector types (e.g., wavelength selectivity for narrowband detectors,^131–140^ operational modes for junction-based detectors,^141–148^ and plasticity for optoelectronic synapses^149–152^ ), as shown in Figure 3b. A thorough understanding of these structures and performance metrics is essential for appreciating how tunable figures of merit can enhance device performance and unlock new functionalities such as in-sensor computing and computational spectrum reconstruction (Figure 3c). This section focuses on the fundamental architectures and tunable parameters of photodetectors, while novel devices with more complex hybrid structures can be found in recent focused reviews.^42,49,50,153–160^

**Figure 3:**
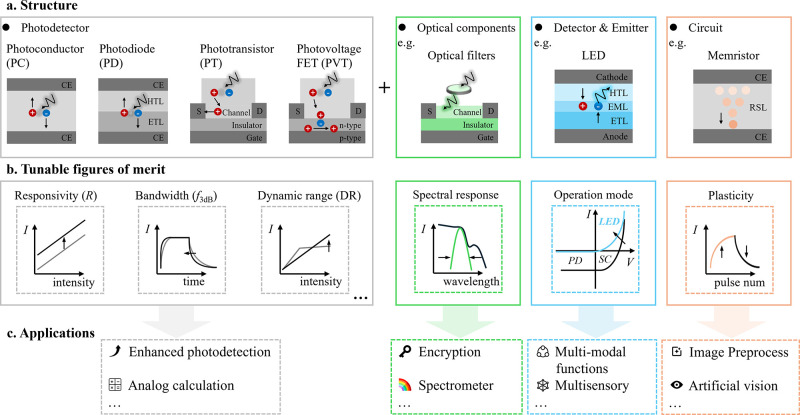
Overview of intelligent photodetectors. (a) Typical photodetector structures and examples of optoelectronic components that can be integrated, defining the tunable performance metrics of intelligent photodetectors. CE: conductive electrode; ETL: electron transport layer; HTL: hole transport layer; FET: field-effect transistor; EML: emissive layer; RSL: resistive switch layer. (b) Examples of tunable performance metrics, showcasing how postmanufacturing tunability enhances photodetection performance and enables advanced functions. (c) Examples of advanced functions discussed in this review.

### 2.1 Structure of Intelligent Photodetectors

#### 2.1.1 Photoconductor

Photoconductors represent the simplest form of two-terminal photodetectors in which a semiconductor is sandwiched between a pair of ohmic contacts. When exposed to light, the semiconductor absorbs photons and generates excess carriers, thereby enhancing conductivity and producing a measurable photocurrent when a bias voltage is applied.^161^ A key advantage of photoconductors is their photoconductive gain, wherein carriers can circulate multiple times through the device before recombination, often yielding an external quantum efficiency (EQE, defined as the number of charge carriers collected from the device per incident photon) exceeding 100%.^162^

Despite their high gain, photoconductors are prone to high dark current due to the absence of a built-in electric field and slower response speed due to the prolonged carrier lifetimes. These characteristics, conventionally considered as drawbacks of photoconductors, can be utilized in intelligent photodetectors to generate synaptic behaviors (to be discussed in Section 4).^49^ However, phototransistors are generally more preferable for applications requiring a higher degree of modulation freedom.

#### 2.1.2 Photodiode

Photodiodes rely on p-n, p-i-n, or Schottky junction architectures to convert absorbed light into electrical signals via a photovoltaic effect. In these devices, the built-in electric field separates photogenerated electron–hole pairs, thereby producing a photocurrent. When operated under reverse bias, photodiodes can achieve high-speed performance by accelerating carrier transit.^162–165^ In photovoltaic mode, EQE of photodiodes is typically below 100%, as each photon generally produces only one electron–hole pair. However, specialized photodiodes works in other modes, such as avalanche photodiodes (APDs) and photomultiplication-type organic photodiodes (PM-OPDs),^166–169^ can still achieve EQE exceeding 100% through various carrier multiplication mechanisms.

Photodiodes are an attractive platform for intelligent photodetectors due to the inherent tunability of their junctions. For instance, the depletion region width can be modulated by adjusting the bias voltage, enabling spatial control over the photocarrier dynamics. This capability is crucial for achieving tunable spectral response, as discussed in Section 5. Furthermore, the versatility of p-n and p-i-n junctions allows these devices to switch among multiple operational modes—including functioning as a photodetector, solar cell, or LED—as illustrated in Figure 3b.

#### 2.1.3 Phototransistor

Phototransistors, often configured as field-effect transistors (FETs), use the channel as both the light-absorbing layer and the conduction pathway. In standard FETs, the gate voltage controls the carrier concentration in the channel, modulating its conductivity. Similarly, in a phototransistor, absorbed photons generate additional carriers, modulating the channel’s conductance and altering the drain current—much like applying an effective gate voltage. A key advantage of phototransistors is their ability to achieve high external quantum efficiency (EQE), often exceeding 100%, due to transconductance and photoconductive gain, making them ideal for detecting weak light signals.^18,170–173^

The three-terminal configuration of phototransistors allows independent control of source and gate voltages, enabling fine-tuning of responsivity, bandwidth, noise, etc. This tunability is essential for in-sensor computing and neuromorphic applications, where the device can adapt its amplification and temporal response dynamics to varying light conditions and task requirements.

#### 2.1.4 Photovoltage Transistor

The photovoltage transistor (PVT) represents a novel hybrid architecture (Figure 3b) that integrates the photovoltaic effect with FET operation.^130,174–176^ It uses electrostatic effects, similar to a junction-gated FET, to modulate charge transport in the channel. In a PVT, the interaction between the light-absorbing layer and the charge transport channel generates a photovoltage upon light illumination, acting like a gate voltage in traditional FETs. This photovoltage itself directly controls charge carrier flow in the channel, which is different from conventional phototransistors relying on photogenerated carrier transport for gain. This unique mechanism allows for precise control of the carrier dynamics. Currently, PVTs are mainly employed in high-sensitivity imaging, and their three-terminal configuration along with a logarithmic response to light intensity show promise for a range of advanced intelligent applications.

### 2.2 Tunable Figures of Merit of Intelligent Photodetectors

#### 2.2.1 Dark Current, Photocurrent and Conductivity

Dark Current (*I*_D_) is the current that flows through a photodetector in the absence of light. It arises from thermally generated carriers and leakage currents in the device and is a critical parameter, as it contributes to the noise. High dark current is generally undesirable because it reduces the signal-to-noise ratio (SNR) and limits the detector’s ability to distinguish low-intensity light signals.^177–180^ Photocurrent (*I*_ph_), on the other hand, is the current generated by light absorption. It is usually measured as the difference between the current measured under illumination (*I*_L_) and dark current, i.e., *I*_ph_ = *I*_L_ – *I*_d_. The magnitude of the photocurrent depends on the intensity of the incident light, as well as the responsivity of the photodetector, which will be explained in the next session. In intelligent photodetectors, tunable dark current and photocurrent is usually used as a compensation method for voltage-controlled denoising and scotopic/photopic adaptation.

#### 2.2.2 Responsivity, Quantum Efficiency and Gain

Responsivity (R), which describes the efficiency of photodetectors, is defined as the output current per incident optical power measured in units of A/W. Alternatively, external quantum efficiency (EQE), defined as the ratio of carrier flux to incident photon flux, also serves as a key performance indicator. EQE provides insight into the effectiveness of a photodetector in converting incident photons to charge carriers, thereby influencing the overall responsivity. Internal quantum efficiency (IQE) takes into consideration of absorbance and is sometimes used interchangeably with gain (*G*), which quantifies the number of photoelectrons produced per absorbed photon. In an ideal scenario, each absorbed photon releases exactly one photoelectron, resulting in a gain of 1. However, real-world photodetectors, such as photoconductors and photodiodes, often exhibit gains that are less than 1 due to electron–hole recombination processes. Alternatively, in the presence of traps, the gain can exceed 1, calculated as $G=\frac{\tau_{\mathrm{trap}}}{\tau_{\mathrm{transit}}}$ , where τ_trap_ represents the trapping time and τ_transit_ is the transit time of carriers through the device.^181–183^ These gain-related metrics, represented by responsivity, play vital roles in intelligent photodetectors, particularly in applications involving weight-based multiplication operation.

#### 2.2.3 Response Speed and Bandwidth

Response speed is a critical parameter in photodetectors, determining how quickly the device’s output current reacts to changes in incident light. Response speed is influenced by factors, such as carrier mobility, device design, and signal processing circuits. The response time quantifies the speed at which the photocurrent changes, typically measured as the time required for the photocurrent to shift between 10% and 90% of its final value after a change in light intensity. A shorter response time indicates the photodetector’s ability to quickly follow light variations, making it ideal for high temporal resolution tasks.

The cutoff frequency (*f*_3dB_) is another important aspect, representing the frequency at which the photodetector’s output signal drops to half its low-frequency amplitude (approximately −3 dB). This cutoff marks the point where the device’s response starts to decline due to internal limitations like capacitance (*C*) and transit time (*t*_tr_). In addition to direct measurement, this value can also be derived mathematically, as demonstrated below:^24^

$$f_{-3dB}^{2}={\left(\frac{3.5}{2\pi t_{tr}}\right)}^{2}+{\left(\frac{1}{2\pi RC}\right)}^{2}$$

In dispersive materials, like organic semiconductors, the transit time of the slower carrier dictates the frequency response.^184^ While bandwidth-related metrics like response speed and cutoff frequency are vital in traditional photodetectors, their use in intelligent photodetectors is more limited, primarily in applications that simulate slow adaptive processes or serve as encoders for spiking neural networks. Devices with tunable variable bandwidth can also function as switchable photodetector and optoelectronic synapse.^185,186^

#### 2.2.4 Noise Equivalent Power, and Specific Detectivity

The Noise Equivalent Power (NEP) is the amount of incident optical power (typically measured in watts) that produces a signal equal to the noise level of the photodetector. Essentially, it represents the minimum detectable power that the photodetector can sense with a signal-to-noise ratio (SNR) of 1. A lower NEP indicates a more sensitive photodetector as it can detect weaker signals above the noise level.

Specific detectivity (D*) is an overall figure of merit that normalizes NEP with respect to its area (*A*) and the bandwidth (*B*) which it operates, typically measured in Jones (where 1 Jones = 1 cm·Hz^1/2^W^1–^). It quantifies how effectively a photodetector can detect weak signals, accounting for the detector’s size and bandwidth. Specific detectivity is mathematically defined as the reciprocal of NEP (i.e., SNR) when the detection bandwidth is 1 Hz and the device area is 1 cm^2^ at an incident power of 1W:^20^

$$D^{*}=\frac{\sqrt{AB}}{NEP}=\frac{R\sqrt{AB}}{I_{N}}$$

where *A* is illumination area, *B* is bandwidth, *R* is responsivity, and *I*_N_ is the noise current. A higher detectivity value indicates greater sensitivity to weak signals, which is essential for low-light imaging. It is noteworthy that many sources can contribute to the noise current, including thermal, shot, flicker (1/*f*) and generation–recombination noise.^187–189^ Directly inferring *I*_N_ from the shot noise (simply defined by the dark current) and thermal noise can underestimate noise and therefore overestimate detectivity in disordered semiconducting systems such as organic semiconductors, metal-halide perovskites and colloidal quantum-dots (CQDs).^24^ While NEP and D* are vital metrics for evaluating conventional photodetector performance, they are less frequently reported in intelligent photodetectors due to the difficulty in controlling device noise. However, certain applications, such as random noise generation, may still benefit from noise-related performance metrics.

#### 2.2.5 Dynamic Range, Linear Dynamic Range and Linearity

Dynamic range (DR) is a critical parameter for photodetectors, defining the range of light intensities that the device can accurately measure. It represents the ratio between the maximum and minimum detectable optical power levels that the photodetector can handle, while maintaining a linear response. A wider dynamic range allows the photodetector to operate effectively across a broader spectrum of light intensities, from very dim to very bright conditions.

The dynamic range is typically expressed in decibels (dB) and can be calculated as

$$DR=20\;\log\left(\frac{P_{\max}}{P_{\min}}\right)$$

where *P*_max_ is the maximum detectable power before the detector saturates, and *P*_min_ is the minimum detectable power above the noise floor. A high dynamic range is essential in applications where the light intensity varies significantly. Therefore, tunable DR is frequently witnessed in scotopic/photopic adaptation. An adaptive dynamic range allows the sensor to capture details in both dark and bright areas of a scene without losing information to saturation or noise.

Specifically, the linear dynamic range (LDR) refers to the range of light intensities where the photocurrent (*I*_ph_) is linearly proportional to the light intensity (*P*), typically expressed as *I*_ph_ ∝ *P*. In some photodetectors, particularly those with carrier traps, achieving a fully linear photoresponse may not be possible. In such cases, a dynamic range where the photocurrent and light intensity follow a logarithmic relationship, i.e., log *I*_ph_ ∝ log *P*, can be considered acceptable for defining the LDR. Under these conditions, the relationship between photocurrent and light intensity takes the form *I*_ph_ ∝ *P* ^α^, where α describes the linearity. The closer α is to 1, the more linear the photoresponse. Photodetectors based on two-dimensional materials (2DMs) typically show a sublinear power-law dependence (0 < α < 1), which is attributed to the complex processes of carrier generation, trapping, and recombination occurring within these detectors.^190,191^

#### 2.2.6 Spectral Response

Spectral response of photodetectors refers to its sensitivity to incident light of different wavelengths. This parameter is crucial as it tells the spectrum regime that a photodetector can respond to and how efficiently it converts light of specific wavelengths into an electrical signal. A photodetector’s spectral response is typically described by responsivity as a function of wavelength, or EQE spectrum (i.e., EQE versus wavelength).

The spectral response is critical in applications that require the detection of specific wavelengths or a broad range of wavelengths. For example, to achieve color vision, photodetectors (or imaging systems) must be able to distinguish between different bands of light. Small full width at half maxima (fwhm; <100 nm) are preferred for these narrowband applications.^138,192–196^ On the other hand, photodetectors with broadband spectral response are capable of acquiring rich features from different spectrum regime.^197–202^ Some devices may even show switchable narrowband and broadband response.^203^ In intelligent photodetectors, tunable wavelength selectivity is usually achieved with electrical modulation^204^ and facilitates applications such as optoelectronic logic gate and reconstructive spectrometer.

#### 2.2.7 Operation Mode

The operation mode is typically not considered as a figure of merit for photodetectors, but in intelligent photodetectors, it becomes crucial for enabling switchable functions after manufacturing. This refers to the ability to switch between modes, such as photodetector, solar cell, or LED, based on external conditions like applied voltage or illumination. In the photodetector mode, the device detects light and converts it into an electrical signal, operating under reverse bias. In solar cell mode, the device converts light into electrical energy under zero or slight forward bias, focusing on the power output for energy harvesting. In LED mode, the device emits light when forward biased, which is useful for displays, lighting, and indicators. This multifunctionality can enhance the efficiency of integrated optoelectronic systems and reduce the need for multiple components.

#### 2.2.8 Plasticity

Similar to operation regime, plasticity is a borrowed concept from neuromorphic devices.^205–210^ It refers to the ability of the device to adapt its response based on past stimuli or environmental conditions, much like synaptic plasticity in biological systems where the strength of connections between neurons changes with experience. In photodetectors, plasticity enables the device to “learn” from previous light exposures, allowing for more complex and dynamic functionality. Plastic photodetectors can retain information about previous light exposures, which influences their responses to future stimuli. This memory effect is crucial for applications like neuromorphic computing, where the device needs to simulate neural processes by adjusting its response based on preprogrammed weights. Meanwhile, Plasticity allows photodetectors to adapt to varying light conditions, improving the performance in environments with fluctuating light intensities by smoothing out random noise. In intelligent photodetectors, plasticity is most related to analogy calculations involving integral operations or mimicking the memory functions of human visual systems.

## 3 Performance Enhancement in Intelligent Photodetectors

After understanding the common structure and performance metrics of photodetectors, we now explore how various tuning mechanisms can be employed to enhance the photodetection performance. As mentioned before, tunability in intelligent photodetectors is not solely determined by the device design but also critically depends on how the device is operated. In this context, photodetectors built upon conventional architectures can also be operated intelligently. To illustrate this concept, we will first describe how each parameter can be dynamically tuned in conventional photodetectors, and then highlight recent innovative device designs that employ postmanufacturing tuning schemes to further enhance photodetection performance. This section focuses primarily on improving key figures of merit, including responsivity, response speed, dynamic range, and signal-to-noise ratio. Additional metrics that extend beyond traditional photodetection, such as the spectral response and plasticity, will be discussed in subsequent sections as we delve into the new functionalities enabled by intelligent photodetectors.

### 3.1 Enhanced Responsivity

Postmanufacturing tunability of responsivity is a straightforward yet powerful example showing the benefit of intelligent photodetectors, as it enables dynamic adjustment of sensitivity based on the intensity of the target light signal.

For instance, in photoconductors, increasing the bias voltage raises the photocurrent (according to the Ohm’s Law) and enhances photoconductive gain (due to accelerated carrier transit).^130^ In photodiodes, applying a reverse bias improves photocarrier collection efficiency, especially when the active layer thickness is comparable to the diffusion length of charge carriers.^139,211,212^ Phototransistors are also well-known for its gate-tunable responsivity, which is achieved by modulating the carrier accumulation and depletion in the channel.^181^

Beyond these conventional designs, tunability in responsivity extends to novel architectures like the photovoltage transistor (PVT) reported by Zhao et al. (Figure 4a).^108^ In the PVT design, the sensing and amplification functions are separated: a PV subcell generating photovoltage upon light illumination, and a FET subcell outputting the electrical signal with gain (Figure 4b). Unlike the photoconductive gain that often comes at the cost of slower response,^162,168,181,211,213,214^ the gain from the FET’s transconductance does not compromise speed, thereby simultaneously achieving high responsivity and fast response (Figure 4c). The responsivity of this PVT is tunable in runtime through the gate voltage, which controls both the bias applied on the photodiode and FET’s transconductance (Figure 4d). Based on the similar concept and device design, researchers have demonstrated responsivity enhancement in PVTs based on Ge/Si,^215^ perovskite,^216^ quantum dots,^217,218^ etc.

**Figure 4:**
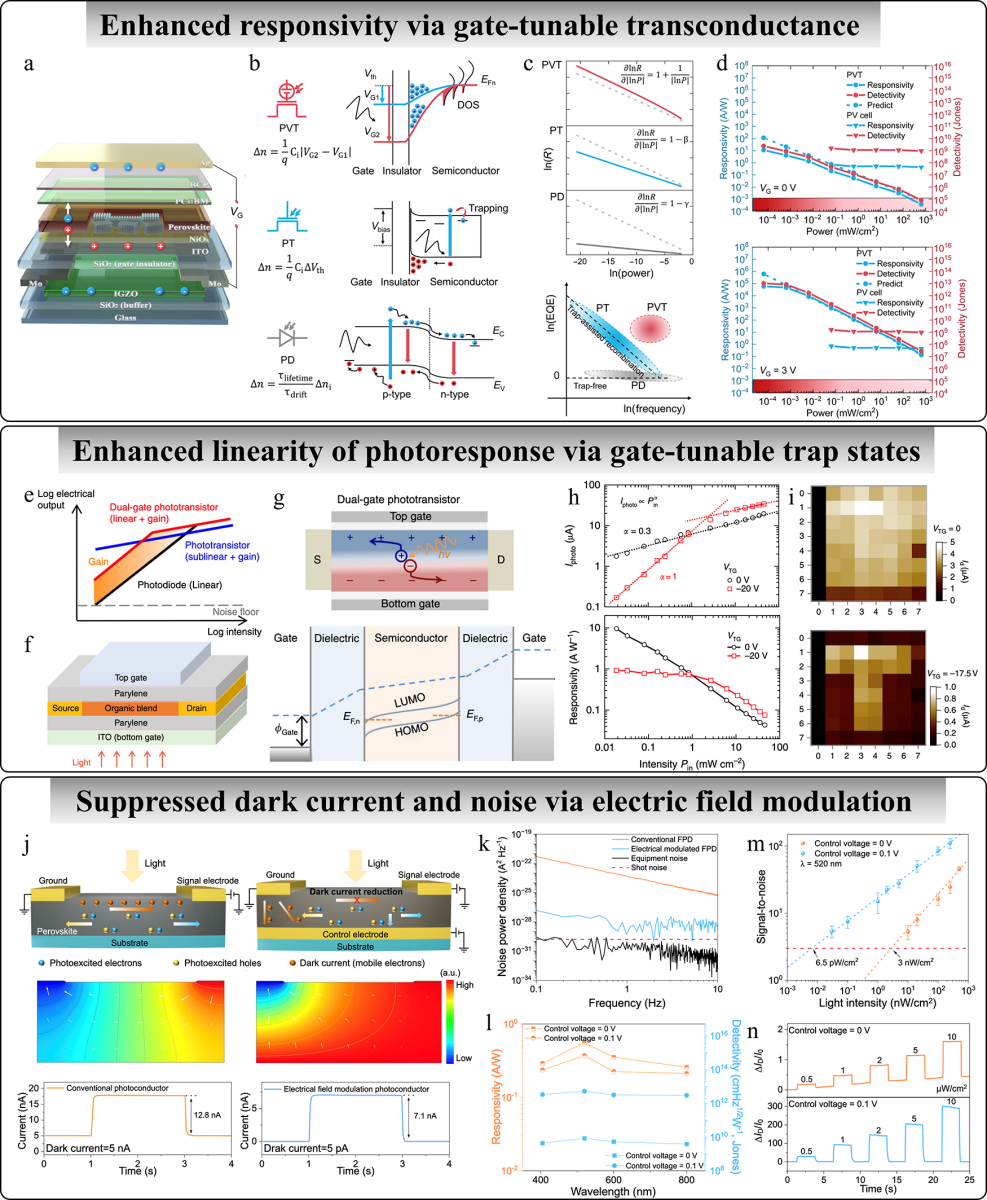
Intelligent photodetectors for enhanced photodetection performance. (a) Schematics of photovoltaic transistor. (b) Comparison between working principles of different types of photodetectors. (c) Comparison of the responsivity–power relationship and EQE-frequency relationship of different types of devices. (d) Light intensity-dependent responsivity and detectivity under 0 and 3 V gate bias. Adapted with permission from ref 108. Copyright 2022 The American Association for the Advancement of Science. (e) Dynamic range and gain of different photodetectors. (f) Device structure. (g) Schematic illustration and band diagrams of dual-gate phototransistor. (h) Light intensity-dependent photocurrent and responsivity. (i) Improved imaging contrast by the top-gate modulation. Adapted with permission from ref 225. Copyright 2015 The Author(s) under Creative Commons Attribution 4.0 International License (https://creativecommons.org/licenses/by/4.0/). (j) Comparison between conventional photoconductor and the photoconductive-type device modulated by a control electrode. (k–n) Comparison of noise, responsivity, detectivity, signal-to-noise ratio, and baseline drift between conventional device and electrical field-modulated device. Adapted with permission from ref 232. Copyright 2023 The Author(s) under Creative Commons Attribution 4.0 International License (https://creativecommons.org/licenses/by/4.0/).

Despite the effectiveness of these tuning approaches, it is important to note that the aforementioned tuning schemes typically come with the drawback of an elevated dark current. Since shot noise ( $I_{n}=\sqrt{2eI_{dark}}$ ) is a significant contributor to the overall noise, higher dark current levels lead to increased noise. In other words, current tuning approaches often face a trade-off between high responsivity and low noise/power consumption. Nonetheless, the tunability in responsivity has already offered the advantage of partially eliminating the need for a preamplifier, thereby simplifying the system integration.

To overcome the limitations associated with dark current, novel mechanisms for enhancing responsivity while maintaining low noise levels must be explored. A promising approach was demonstrated by Tang et al., where in situ electric field modulation was used to “dope” mercury telluride colloidal quantum-dots, forming p–n junctions.^219^ This strategy simultaneously enhanced responsivity and suppressed dark current, offering a viable path toward improved performance in intelligent photodetectors.

### 3.2 Enhanced Response Speed

Achieving faster response speed is critical for applications such as optical communication and vital signal monitoring.^108,130,220,221^ In conventional photodiodes, applying a reverse bias increases the electric field intensity across the depletion region, accelerating the transit of the photogenerated carriers. Moreover, improving the reverse bias also reduces the junction capacitance, lowering the RC time constant and further contributing to a faster response.

Tunable response speed can also be achieved in phototransistors by gate-modulation. For instance, in the presence of carrier traps in the channel, applying a gate voltage that accumulates the carriers can eliminate the trapping effect and significantly enhancing response speed.^222,223^ Alternatively, in hybrid phototransistors deploying a heterostructure as the channel, the gate voltage can modulate the band offset. Adjusting the gate voltage in these devices can optimize carrier transport and recombination, leading to faster response speeds.^222^

Interestingly, the same tunable bias mechanisms can be leveraged inversely to slow the photoresponse or generate persistent photoconductivity, which are crucial features for neuromorphic sensing and will be further discussed in Section 4.

### 3.3 Enhanced Linearity

Linearity is critical for photodetection, ensuring that the output electric signal accurately reflects the intensity of the incident light. Photodiodes are typically favored for their high inherent linearity and wide LDR. Moreover, applying a reverse bias further enhances the linearity by more efficiently sweeping out photocarriers.^224^

While photodiodes exhibit a linear response, they lack intrinsic amplification capability. In contrast, phototransistors can offer photoconductive gain through carrier trapping, but this often results in sublinear photoresponse (Figure 4e). To achieve both sensitive and linear photoresponse simultaneously, Someya et al. developed a dual-gate organic phototransistor that combines the benefits of photodiodes and phototransistors (Figure 4f).^225^ In this design, oppositely biased gate electrodes create n and p channels within the semiconducting layer, establishing a vertical electric field similar to the built-in field in diodes, which separates photogenerated electron–hole pairs (Figure 4g). The top gate voltage (*V*_TG_) adds an extra degree of control over the photodetection performance by modulating carrier concentration and trap states. At *V*_TG_ = 0 V, photogenerated holes become trapped, leading to a photoconductive gain and sublinear photoresponse (Figure 4h). However, applying a negative *V*_TG_, fills the hole traps, allowing photogenerated holes to be extracted and producing a gain independent of light intensity within a certain intensity regime (Figure 4h). By adjustment of *V*_TG_, the authors were able to image a T-shaped pattern in the presence of disruptive full-frame illumination with significantly improved contrast (Figure 4i). Tunable linearity has also been demonstrated with 2D materials with the similar dual-gate phototransistor architecture.^226^

It is important to note that enhancing linearity via dual-gate tuning typically comes at the expense of responsivity (Figure 4h). In practice, this tunability allows another choice: sacrificing linearity, when necessary, to detect weak light signals. Despite its importance, few studies have directly addressed runtime tunable linearity, as most efforts have focused on material optimization and band-structure engineering to maintain a constant high linearity.^227–229^ Future work should aim to develop strategies that enable runtime tunable linearity, thereby broadening the functional scope of intelligent photodetectors.

### 3.4 Suppressed Dark Current and Noise

Suppressing dark current is another challenge for photodetection, as it directly affects the noise level, detectivity, and overall signal-to-noise ratio. In phototransistors, dark current can be effectively reduced through gate voltage modulation or ferroelectric gating, offering a straightforward tuning mechanism.^230,231^ However, the issue can be particularly pronounced in photoconductor-based photodetectors, where the absence of a built-in electric field leads to a higher dark current. While increasing the resistivity of the active layer can help reduce dark current, it also lowers the photocurrent, thereby reducing responsivity and offering only limited improvements in SNR.

To address this challenge, Yang et al. introduced an innovative design incorporating a control electrode that diverts dark current away from the signal electrode (Figure 4j).^232^ By fine-tuning the control electrode voltage, the signal electrode can selectively capture photocurrent while minimizing the dark current. This approach effectively reduces noise without severely affecting photocurrent, enhancing detectivity and improving the signal-to-noise ratio (Figure 4k–n). Additionally, the control voltage reshapes the internal electric field, suppressing ion migration and reducing baseline drift (Figure 4j). This technique has also been successfully implemented in perovskite-based X-ray detectors^233^ and quantum dot-based infrared detectors.^234^

While minimizing dark current is universally desirable for conventional photodetection tasks, most existing studies have focused on material optimization, band structure engineering, interfacial modification, etc.^235–237^ In contrast, relatively few studies have explored postmanufacturing tuning methods for dark current suppression. This approach represents a promising research direction, as it could reduce fabrication complexity and offer additional benefits—such as enabling pixel-by-pixel tuning in imaging arrays for effective dark frame calibration.

## 4 Advanced Functions of Intelligent Photodetectors with Tunable Temporal Response Dynamics

In the previous section, we explored photodetectors with postmanufacturing tunability for enhanced photodetection performance. These devices can be fine-tuned after fabrication to meet specific requirements, offering a high degree of customization that greatly benefits various imaging tasks. In the following three sections, we will delve into how this flexibility and adaptability extend beyond basic imaging functions to enable advanced capabilities. We will start by examining photodetectors with tunable temporal response dynamics (including tunable responsivity, response polarity, response speed, persistent photoconductance, plasticity, etc.), emphasizing their role in fundamental image preprocessing tasks—such as contrast enhancement and noise reduction—as well as more complex functions such as artificial vision. This discussion will demonstrate how tunable photodetectors are not just passive sensors but also active components that contribute to the processing and interpretation of structured visual information, leading to smarter and more efficient imaging systems.

### 4.1 Image Preprocessing

#### 4.1.1 Contrast Enhancement and Denoising

Contrast enhancement and denoising are fundamental tasks in image preprocessing.^238^ Contrast enhancement amplifies the luminance difference between objects and their backgrounds, making features more distinct, while denoising removes unwanted noise to improve image clarity and quality. Although their objectives differ—contrast enhancement highlights features, while denoising reduces background noise—both aim to optimize the signal-to-noise ratio and are often performed simultaneously. This section explores how postmanufacturing tunability in intelligent photodetectors facilitates these processes.

We begin with devices that rely on electrical tuning. Photodetectors such as phototransistors and photodiodes can be tuned by adjusting the applied voltage, allowing for customizable performance during operation. This tunability can therefore be used to compensate for imperfections during the photodetection. For example, Ma et al. demonstrated that by adjusting the gate voltage in a wafer-scale transistor array based on 2D monolayer molybdenum disulfide (MoS_2_),^239^ uniform output currents could be achieved despite fabrication inconsistencies, effectively enhancing image contrast and reducing noise, including salt-and-pepper noise, through voltage-controlled tuning. Additionally, Dodda et al. used electrical programmability to address the memory effect in MoS_2_ transistors,^240^ which can lead to persistent high conductance due to random photoexcitation. By applying a positive gate voltage, they achieved fast resets and denoising, enabling accurate image capture under noisy conditions. Furthermore, in-sensor filtering via convolution operations, a method similar to edge detection, presents another approach to image denoising.^241^ This technique will be explored further in Section 4.1.2.

While electrical tuning provides a degree of postmanufacturing adaptability for image preprocessing, it typically requires peripheral circuits or preset control schemes to determine the applied voltage, thereby limiting the device’s ability to autonomously adapt to changes in illumination. For self-enhancement or denoising, light itself should guide the device’s modulation by leveraging the information contained in the incident light. Optoelectronic synapses are ideal for such in-sensor image preprocessing due to their light-tunable plasticity, where current and retention time increase with higher light intensity, helping to accumulate signals and smooth out noise. Zhou et al. introduced a two-terminal optoelectronic resistive random-access memory (ORRAM) device^58^ based on molybdenum oxides (MoO_*x*_), exhibiting nonvolatile optical resistive switching and light intensity-tunable synaptic behavior. The ORRAM arrays can perform image sensing, memory functions, and neuromorphic visual preprocessing like contrast enhancement and noise reduction. When the MoO_*x*_ thin film absorbs UV light, it undergoes a resistance state change due to the formation of hydrogen molybdenum bronze (HyMoO_*x*_) (Figure 5a). This results in time-dependent output currents (Figure 5b), enabling image preprocessing where brighter pixels accumulate signals more effectively, enhancing contrast, while random noise fades naturally as the current diminishes over time (Figure 5c). In addition to directly processing the light intensity, optoelectronic synapses can also use a number of light pulses as input stimulation. Zhai et al. demonstrated an optoelectronic synapse using a defect-rich Fe_7_S_8_ core with a MoS_2_ dome shell (Figure 5d),^242^ showing light-tunable synaptic behaviors (Figure 5e). By encoding grayscale into a number of light pulses, they effectively reduced noise and improved image quality. Based on similar ideas, there are many other works effectively realize the contrast enhancement or “self-denoising” function in optoelectronic synapses.^59–62,250–252^

**Figure 5:**
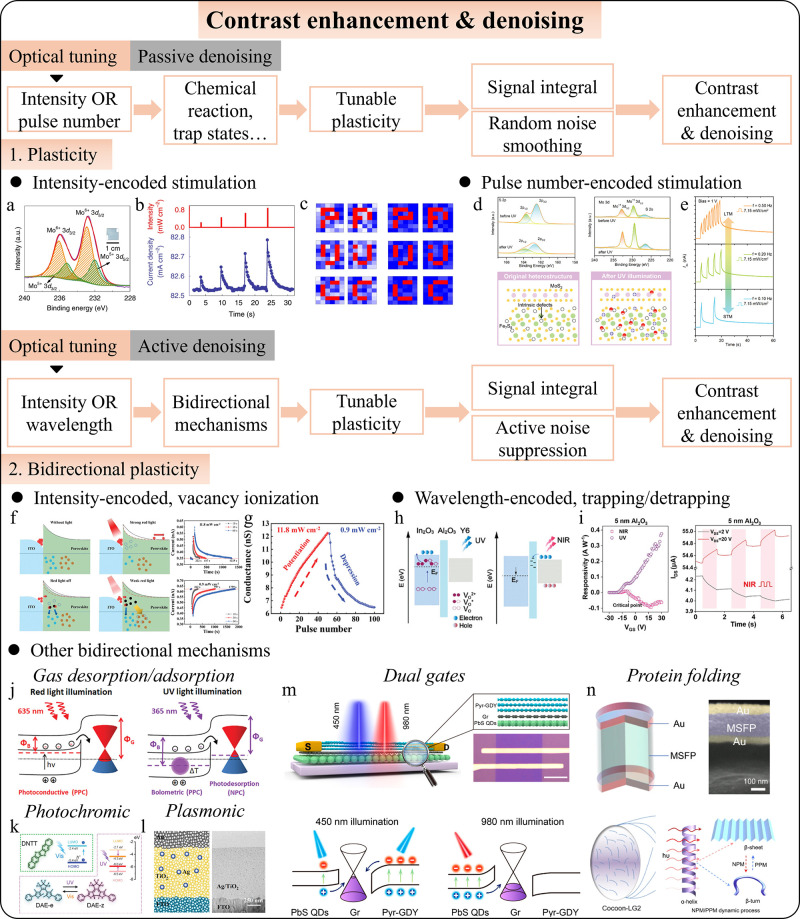
Intelligent photodetectors for contrast enhancement and denoising. (a) UV-induced chemical reaction in the MoO_*x*_-based ORRAM. (b) Light intensity-dependent STP. (c) Demonstration of contrast enhancement. Adapted with permission from ref 58. Copyright 2019 The Author(s) under exclusive license to Springer Nature Limited. (d) Illustration of UV-induced interfacial charge transfer in Fe_7_S_8_@MoS_2_ core–shell structures. (e) Pulse number-dependent photoresponse showing transition from STM to LTM. Adapted with permission from ref 242. Copyright 2024 Wiley-VCH GmbH. (f) Switching mechanism and (g) bidirectional plasticity of the retinomorphic memristor. Adapted with permission from ref 243. Copyright 2023 Wiley-VCH GmbH. (h) Switching mechanism and (i) wavelength-dependent bidirectional plasticity of the In_2_O_3_/Al_2_O_3_/Y6 neuromorphic phototransistors. Adapted with permission from ref 244. Copyright 2023 Wiley-VCH GmbH. (j–n) Different mechanisms to realize tunable potentiation/depression plasticity. (j) Adapted with permission from ref 245. Copyright 2020 WILEY-VCH Verlag GmbH & Co. KGaA, Weinheim. (k) Adapted with permission from ref 246. Copyright 2021 Wiley-VCH GmbH. (l) Adapted with permission from ref 247. Copyright 2021 The Authors. Advanced Science is published by Wiley-VCH GmbH. (m) Adapted with permission from ref 248. Copyright 2020 American Chemical Society. (n) Adapted with permission from ref 249. Copyright 2023 The Author(s) under Creative Commons Attribution 4.0 International License (https://creativecommons.org/licenses/by/4.0/).

While the natural drop in photocurrent (forging process) and spike-based mapping can automatically process images, they often sacrifice efficiency and require extra time for stabilization. For cases needing rapid, high-quality image capture, an active noise suppression method is essential. Bidirectional photoresponse (BPR) synapses address this by using a second light of different intensity or wavelength (often invisible) to suppress noise. Unlike traditional optoelectronic synapses, which only show positive photoresponses, BPR synapses can switch between excitatory and inhibitory behaviors based on the light’s wavelength. Chen et al. demonstrated a perovskite memristor with intensity-dependent BPR based on vacancy ionization (Figure 5f),^243^ where strong light increases conductivity and weak light decreases it (Figure 5g), helping to eliminate image blurring and ghosting. However, using similar wavelengths for signal and noise suppression can be problematic, as simultaneous application can result in strong light that cancels out the suppression effect, requiring separate denoising steps that increase time and energy consumption. Zhu et al. proposed a solution using a wavelength-dependent BPR synapse based on In_2_O_3_/Al_2_O_3_/Y6 phototransistors utilizing trapping/detrapping (Figure 5h),^244^ which utilizes different wavelengths to achieve simultaneous potentiation and depression (Figure 5i). This approach enables active noise reduction with NIR pulses, while maintaining tunable plasticity for contrast enhancement. In addition to vacancy ionization^253^ and trapping/detrapping mentioned above, several other techniques can achieve tunable potentiation/depression in BPR synapses (Figure 5j–n), such as dual gates,^248^ gas desorption/adsorption,^245^ plasmonics,^247^ photochromism,^246^ biological processes like protein folding^249^ and many other mechanisms.^82,91,92,254–257^ These diverse mechanisms expand the potential applications of BPR synapses, paving the way for all-optical tuning capabilities.

#### 4.1.2 Feature Extraction

Feature extraction is a foundational step in image processing and computer vision, wherein raw image data are transformed into a more compact and informative representation that facilitates subsequent analysis and decision-making. This process aims to identify patterns, structures, or regions of interest that capture the essential characteristics of a visual scene.

Edge detection is one of the most widely employed feature extraction techniques that indicate object boundaries and key features.^258^ Traditional edge detection relies on software algorithms like the Sobel operator, Canny edge detector, Prewitt operator, and Laplacian of Gaussian, which are effective but computationally intensive and slow, especially for real-time applications. Intelligent photodetectors offer a more efficient solution by performing in-sensor preprocessing, dynamically adjusting the photoconductivity to simulate traditional algorithmic kernel weights. This reduces redundant data and accelerates the processing. For instance, Wang et al. developed a symmetric n/p/n structure using MoS_2_ and WSe_2_,^259^ achieving in situ edge extraction by arranging a pair of oppositely biased photodetectors (Figure 6a). When both detectors are equally illuminated, their photocurrents cancel each other out, resulting in zero total current. A nonzero current is generated only when the two detectors receives light of different intensity, indicating the presence of an edge (Figure 6b). Beyond simply pairing photodetectors with opposite photoresponse polarity, devices can also be configured to mimic the human retina using artificial retinomorphic vision sensors (Figure 6c).^260^ These sensors simulate the behavior of photoreceptors and bipolar cells by converting light into electrical signals with either positive or negative responses (Figure 6d), depending on the applied gate voltage. By arranging these sensors into arrays, they can replicate the retina’s dynamic light response, using ON and OFF photoresponse devices to detect and process edges in real-time (Figure 6d).

**Figure 6:**
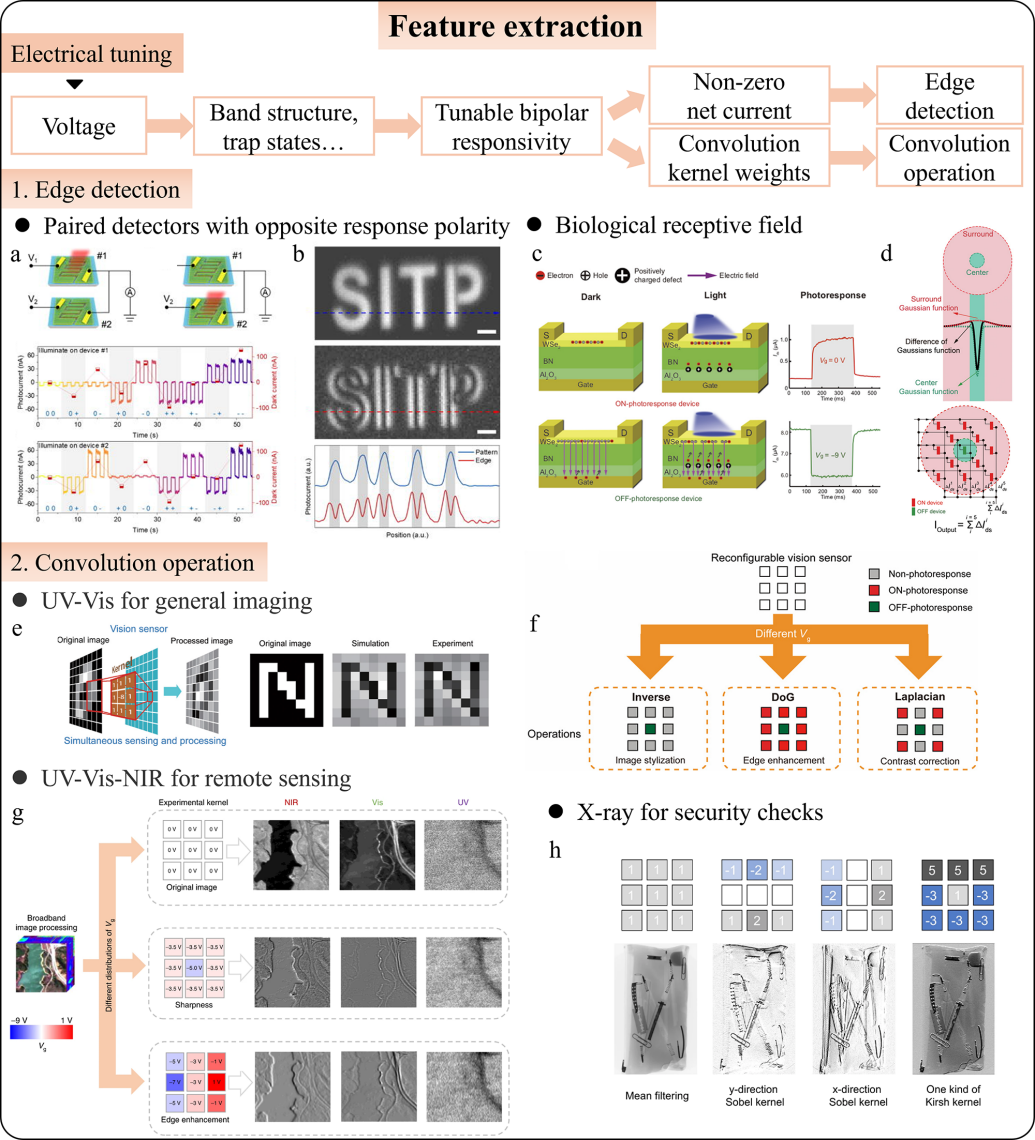
Intelligent photodetectors for feature extraction. (a) Joint photodetection diagram of paired photodetectors. (b) Demonstration of edge extraction. Adapted with permission from ref 259. Copyright 2024 AIP Publishing. (c) Operating mechanism and gate-tunable photoresponse of the retinomorphic device. (d) Demonstration of edge detection. (e–f) Schematics of simultaneous image sensing and convolutional operation. Adapted with permission from ref 260. Copyright 2020 The American Association for the Advancement of Science. (g–h) Convolutional processing of broadband and X-ray images. (g) Adapted with permission from ref 261. Copyright 2022 The Author(s), under exclusive license to Springer Nature Limited. (h) Adapted with permission from ref 262. Copyright 2024 The Author(s) under Creative Commons Attribution 4.0 International License (https://creativecommons.org/licenses/by/4.0/).

Based on the same mechanism of tunable bipolar responsivity, intelligent detectors can perform more generalized convolution operations. As mentioned earlier, edge detection is essentially a type of convolution operation. By applying a sliding convolution kernel to an image, various image processing tasks can be accomplished, extracting not only edges but also textures and patterns by adjusting the kernel’s parameters. Convolution kernels can also be used for tasks such as image inversion and stylization. A notable example of this can be found in the same work on artificial retinomorphic vision sensors finished by Wang et al.^260^ Using van der Waals (vdW) heterostructure devices, the gate voltage (*V*_g_) applied to each unit can encode the weights of a 3 × 3 convolution kernel. As the kernel slides across the image, it processes each 3 × 3 pixel block, generating a new image (Figure 6e). These photodetectors are reconfigurable at runtime, allowing kernel weights to be adjusted for tasks such as image stylization, edge enhancement, and contrast correlation (Figure 6f). By changing the photodetector materials, the detection spectrum can extend to near-infrared (NIR) (Figure 6g)^261^ and X-rays (Figure 6h),^262^ with applications in remote sensing for geological exploration, security screening for prohibited items, and nondestructive analysis of packaged chips and biological cells.^263^ There are many other works also realize hardware implementation of convolutional operations, processing extra information such as polarization.^63–65,241,264^ Future works may explore all-optical modulation, updating kernel weights with nonelectrical methods, enabling the system to adapt to environmental feedback or specific tasks, further enhancing the versatility and efficiency of image preprocessing.

#### 4.1.3 Motion Detection

Motion detection is a common task in scenarios such as visual surveillance, traffic monitoring, and autonomous driving. Traditional motion detection relies on software algorithms, such as frame differencing, background subtraction, and optical flow, to compare consecutive video frames. However, intelligent photodetectors can detect motion directly within the sensor, minimizing redundant data and preserving critical information. Zhou et al. developed a retina-inspired 2D heterostructure device that implements frame differencing in hardware,^265^ using gate voltage-tunable positive and negative photoconductivity (PPC/NPC) (Figure 7a). The device multiplies positive and negative conductance matrices with image pixels over time, summing the results to detect motion. If no motion occurs, pixel brightness remains near zero; motion results in a nonzero output, indicating movement (Figure 7b). To reduce ghosting, they extended the frame difference time, effectively separating moving objects across a wide spectral range (Figure 7c).

**Figure 7:**
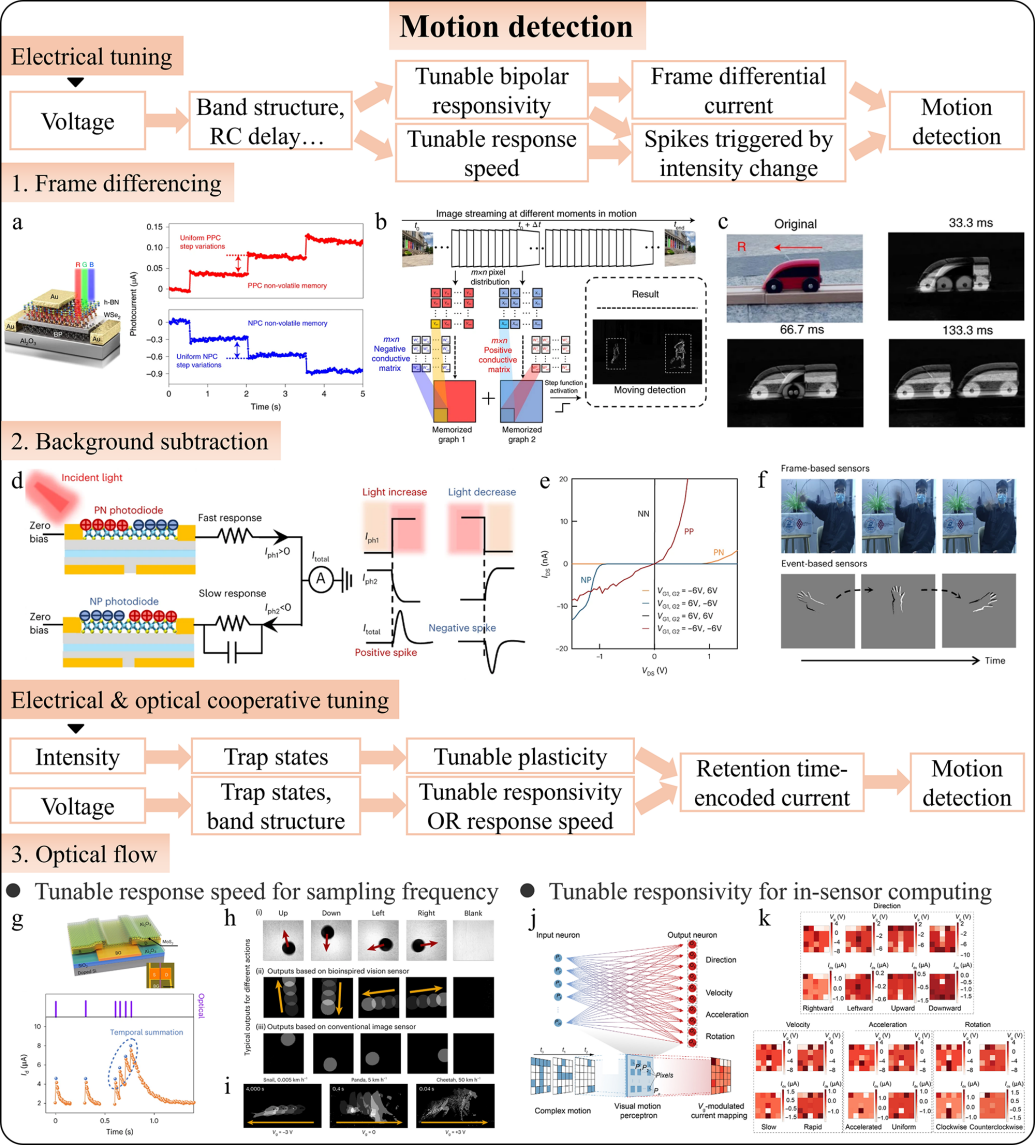
Intelligent photodetectors for motion detection. (a) Device structure and cumulative positive and negative photoconductivity. (b) Illustration of frame differencing. (c) Demonstration of motion detection. Adapted with permission from ref 265. Copyright 2021 The Author(s) under exclusive license to Springer Nature Limited. (d) Illustration of spikes generated by light intensity changes. (e) Tunable polarity and responsivity of the WSe_2_ photodiode. (f) Comparison between frame- and event-based vision sensors. Adapted with permission from ref 266. Copyright 2023 The Author(s) under exclusive license to Springer Nature Limited. (g) Structure and tunable plasticity of MoS_2_ phototransistor. (h) Comparison between output of bioinspired vision sensor and conventional image sensor. (i) Demonstration of capturing motion at different speeds using gate-tunable response speed. Adapted with permission from ref 267. Copyright 2023 The Author(s) under exclusive license to Springer Nature Limited. (j) Schematics of the motion perceptron. (k) Output result of the motion perceptron. Adapted with permission from ref 268. Copyright 2023 American Association for the Advancement of Science.

Despite frame differencing reduces data transmission, static background information still requires storage before subtraction. Event-driven photodetectors, which generate signals only when the light intensity changes, offer a more efficient solution. Zhou et al. reported an event-driven photodetector with PN and NP branches that produce opposite photocurrents with different response times (Figure 7d).^266^ In static scenes, these currents cancel each other out, resulting in a zero output. When the light intensity changes, the branches generate transient spikes, indicating motion. To create a reconfigurable device with switchable PN or NP states (Figure 7e), Chai et al. introduced a modified photodiode structure with two local gates, allowing independent modulation of carrier types and densities. A floating gate structure enabled nonvolatile tuning, adjusting responsivity, and spike signal amplitude. By incorporation of capacitors with different capacitances, they achieved tunable photoresponse times, allowing for programmable event-driven spike signals at sensory terminals. This setup enabled an in-sensor spiking neural network capable of categorizing motion types based on output spiking times (Figure 7f). Unlike frame-based sensors that capture all pixels at a fixed rate, event-driven sensors asynchronously capture only changes in light intensity, significantly enhancing the efficiency and reducing data storage requirements.

While frame differencing highlights moving objects by subtracting static backgrounds (the branch without a transient spike), optical flow provides detailed information on the direction and speed of movement. Conventional optical flow requires continuous image capture and motion vector calculations based on pixel intensity changes. However, intelligent photodetectors with optoelectronic synapses offer a new approach: compressing temporal information into a single image and reducing data processing needs. Chai et al. developed a MoS_2_ phototransistor with light intensity-dependent plasticity,^267^ where intrinsic defects cause trapped photogenerated carriers to create a sublinear increase in conductance (Figure 7g), mimicking neurotransmitter release in neurons. This device encodes motion information as gradually decaying photocurrents (Figure 7h), with gate voltage tuning the response speed from 10^1^ to 10^6^ ms (Figure 7i). The encoded images from this neuromorphic sensor improve motion recognition accuracy by capturing compressive temporal states, unlike conventional sensors that only capture current stimulation.

Miao et al. reported a WSe_2_/h-BN heterostructure device array for motion detection, featuring light intensity-tunable plasticity.^268^ The device’s memory state, induced by light under a negative gate bias, can be gradually erased with pulses, encoding spatiotemporal motion information. The array functions as an in-sensor visual motion perceptron, where gate voltage acts as the perceptron’s weight (Figure 7j), enabling recognition of various motions like “rightward” or “clockwise” (Figure 7k). While optical flow provides detailed motion information, it struggles to filter out background elements, leading to ghosting and inaccurate detection in noisy environments. Future intelligent photodetectors could address this by implementing a threshold mechanism that selectively triggers memory storage, focusing on significant motion, and ignoring background noise. It is noteworthy that several other studies have also introduced innovative solutions for motion detection.^67–71,269,270^ Since some of these works rely specifically on the neuromorphic computing capabilities discussed in Section 4.2.3, we will not discuss them in detail here as image preprocessing techniques.

### 4.2 Artificial Vision

In addition to image preprocessing, artificial vision is another area where intelligent photodetectors outperform conventional photodetectors. While image preprocessing focuses on enhancing image quality and extracting basic features, preparing images for further analysis, artificial vision replicates the complex processing abilities of biological vision systems.^271^ This encompasses high-level tasks such as image memorization, scotopic and photopic adaptation, collision detection, and pattern recognition. Intelligent photodetectors enable real-time understanding and interaction without the need for extensive external computation, significantly enhancing the efficiency and performance.

#### 4.2.1 Image Memorization, Trace Extraction and Selective Attention

Memory is a fundamental function of the human visual system. To simulate this capability, traditional solutions often require additional memory devices to store the information perceived by the image sensor arrays. However, intelligent photodetectors can store visual information directly through in-sensor-tunable conductivity, similar to how the brain retains visual memories. Optoelectronic synapses in these devices retain charge states corresponding to visual inputs, mimicking synaptic plasticity and allowing the device to “remember” images by maintaining photogenerated carrier states, enabling recall and processing without reillumination. Beyond basic memory, intelligent photodetectors can perform functions such as trace extraction and selective attention. Memorized images can create spatiotemporal maps to track object movement, while selective attention focuses on specific regions or features, ignoring irrelevant background information. Some devices even have a wavelength selectivity for extracting relevant signals from complex environments.

The memory capability of intelligent photodetectors is often enabled by the tunable plasticity of optoelectronic synapses, with longer retention times being crucial for effective image memorization. Early examples include a photosensitive structure by Shen et al. that combined a nonvolatile memristor with a UV-sensitive photodetector,^272^ allowing for long-term memory storage without leakage (Figure 8a). More recent devices, like the phototransistor developed by Ahmed et al.,^273^ use bidirectional photoresponse (BPR) to write or erase information with ultraviolet light, achieving precise memory control through oxidation-induced defects in black phosphorus layers (Figure 8b). Given that image memorization is a relatively fundamental function of an optoelectronic synapse, we reserve the discussion for more advanced functions enabled by these memory capabilities.

**Figure 8:**
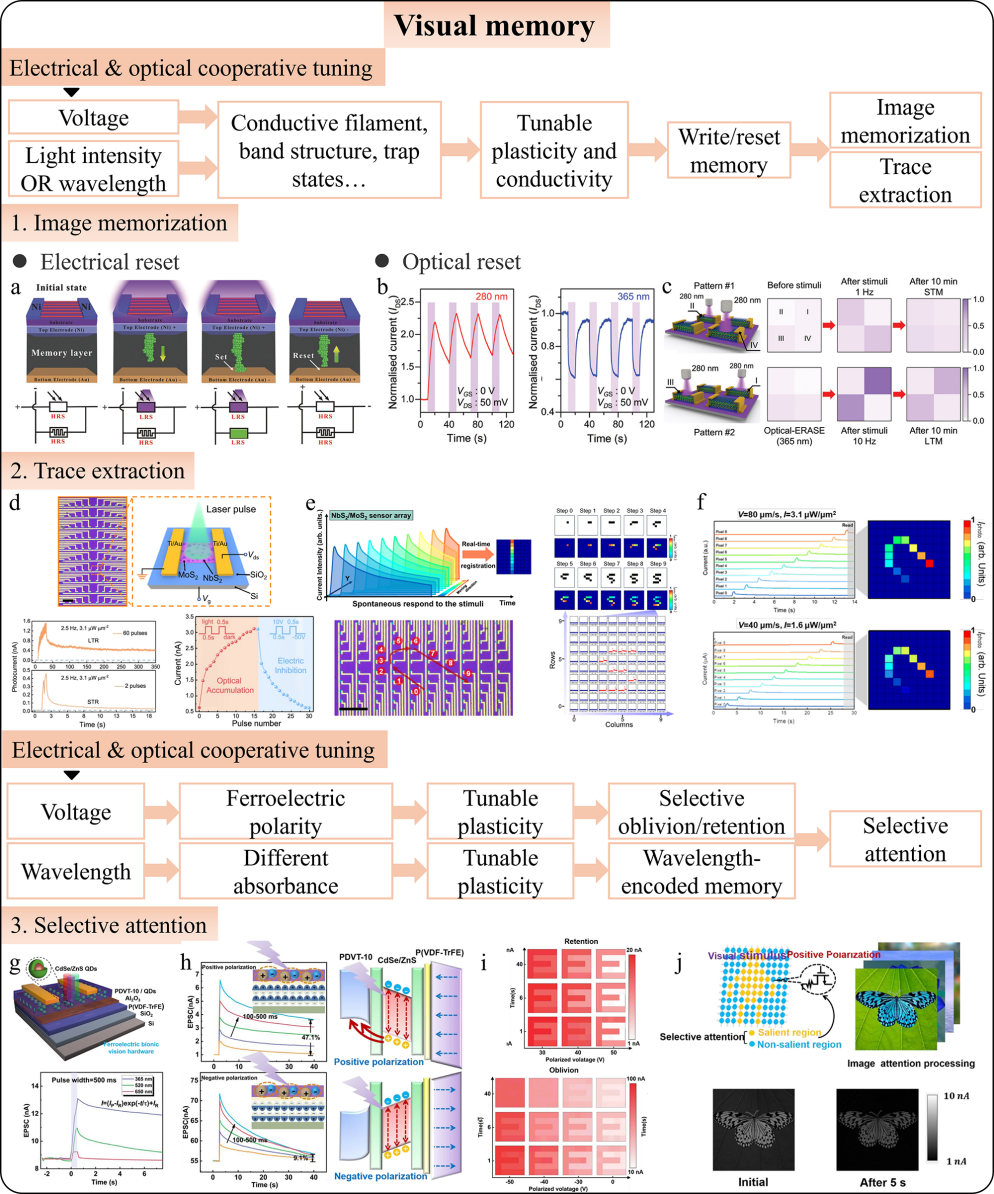
Intelligent photodetectors for visual memory. (a) Schematic illustrations of the device structure and switching mechanism. Adapted with permission from ref 272. Copyright 2018 WILEY-VCH Verlag GmbH & Co. KGaA, Weinheim. (b) Wavelength-dependent bidirectional plasticity of the BP phototransistor. (c) Demonstration of Image detection and memorization. Adapted with permission from ref 273. Copyright 2020 Wiley-VCH GmbH. (d) Device structure and pulse number-dependent plasticity of the NbS_2_/MoS_2_ phototransistor. (e) Demonstration of trajectory registration. (f) Trajectory registration performance under different intensities and velocity. Adapted with permission from ref 274. Copyright 2023 The Author(s) under Creative Commons Attribution 4.0 International License (https://creativecommons.org/licenses/by/4.0/). (g) Device structure and wavelength-dependent plasticity of the ferroelectric phototransistor. (h) Voltage-dependent plasticity and switching mechanism. (i–j) Demonstration of electrical selective attention and optical selective attention. Adapted with permission from ref 275. Copyright 2022 The Author(s) under Creative Commons Attribution 4.0 International License (https://creativecommons.org/licenses/by/4.0/).

Enhancing light tunability along with photoconductivity allows intelligent photodetectors to perform new functions such as trace extraction and selective attention. In these applications, variable retention time is crucial, making trapping-based devices ideal for short- to medium-term data storage. For instance, Xu et al. developed a NbS_2_/MoS_2_ phototransistor with optically tunable plasticity,^274^ where retention time increases with light pulses (Figure 8d). In an image array, these devices can track moving light spots through time-dependent photocurrent decay, effectively registering light trajectories (Figure 8e). However, this method is best suited for constant light intensity and can become complicated if the trace intersects with itself (Figure 8f).

Another application of image memorization is simulating the selective attention of the human visual system.^276,277^ While typical optoelectronic synapses preserve all detected information, the human brain selectively retains objects that receive attention, with unnoticed objects fading from memory. Chen et al. created a ferroelectric device with both electrical and optical selective attention.^275^ Wavelength-dependent plasticity allows shorter wavelengths to induce higher conductivity and longer retention times (Figure 8g). Under the same wavelength, positive or negative voltage modulates the device’s retention state. Positive polarization extends memory retention (i.e., retention process), while negative polarization leads to rapid memory loss (i.e., oblivion process) (Figure 8h). This allows selective memorization of light information, where, for example, in a complex image only regions illuminated under positive voltage are remembered, while others quickly fade (Figure 8g). Additionally, patterns with shorter wavelengths, like a blue “butterfly”, are retained longer, while features like a green “leaf” disappear within seconds (Figure 8h). In addition to the examples discussed above, many other applications can be implemented utilizing the image memorization capability, such as emotional simulation, skin sunburned simulation and conditional reflex behavior.^72–74,278–284^

#### 4.2.2 Scotopic/Photopic Adaptation

Scotopic and photopic adaptations enable vision across a wide range of lighting conditions from dim starlight to bright sunlight. Scotopic vision, governed by rod cells, adapts to low light, while photopic vision, controlled by cone cells, adjusts to bright light. Replicating these natural adaptations in artificial vision systems is crucial for the development of versatile and efficient imaging technologies. Intelligent photodetectors, with their real-time processing and adaptive capabilities, show promise in integrating these adaptations. Current research focuses on two main approaches: feed-forward and feedback stimulation. Feedback involves the brain adjusting early visual processing, modulating sensitivity, and contrast through voltage tuning based on photodetector signals. Feedforward mimics the direct transmission of visual information, allowing photodetectors to automatically adapt without a higher system involvement. Both approaches use strategies to either compensate existing signals (i.e., postreceptoral adaptation) or alter the intrinsic photoresponse (i.e., photoreceptor adaptation), such as responsivity or dynamic range.

Park et al. developed an early example of scotopic and photopic adaptation using an optoelectronic circuit with a photodetector,^285^ load transistor, and synaptic transistor (Figure 9a). The circuit adjusts photovoltage signals by varying the gate voltage on the load transistor, enhancing sensitivity in low light (scotopic adaptation) or suppressing signals in bright light (photopic adaptation; Figure 9b). As technology developed, photodetection was integrated directly into optoelectronic synapses. Liao et al. demonstrated a MoS_2_ phototransistor with voltage-controlled trapping/detrapping processes (Figure 9c),^286^ enabling adaptive vision in varying light conditions (Figure 9d). Many other works realize scotopic and photopic adaptation based on similar ideas of compensation current.^76,78,80,81,290,291^ and can easily extend-capabilities to polarization adaptation.^292,293^ Besides, adaptations also apply to spiking signals, which are discrete and encode illumination levels in the time-to-first-spike. Das et al. introduced an adaptive photoencoder with time-delayed spiking,^294^ where brighter light triggers earlier spikes and dimmer light delays them, mimicking the plasticity of human vision. The encoder adjusts to different lighting conditions by reprogramming the threshold voltage, making it an effective tool for converting sensory stimuli into spike trains across various lighting environments.

**Figure 9:**
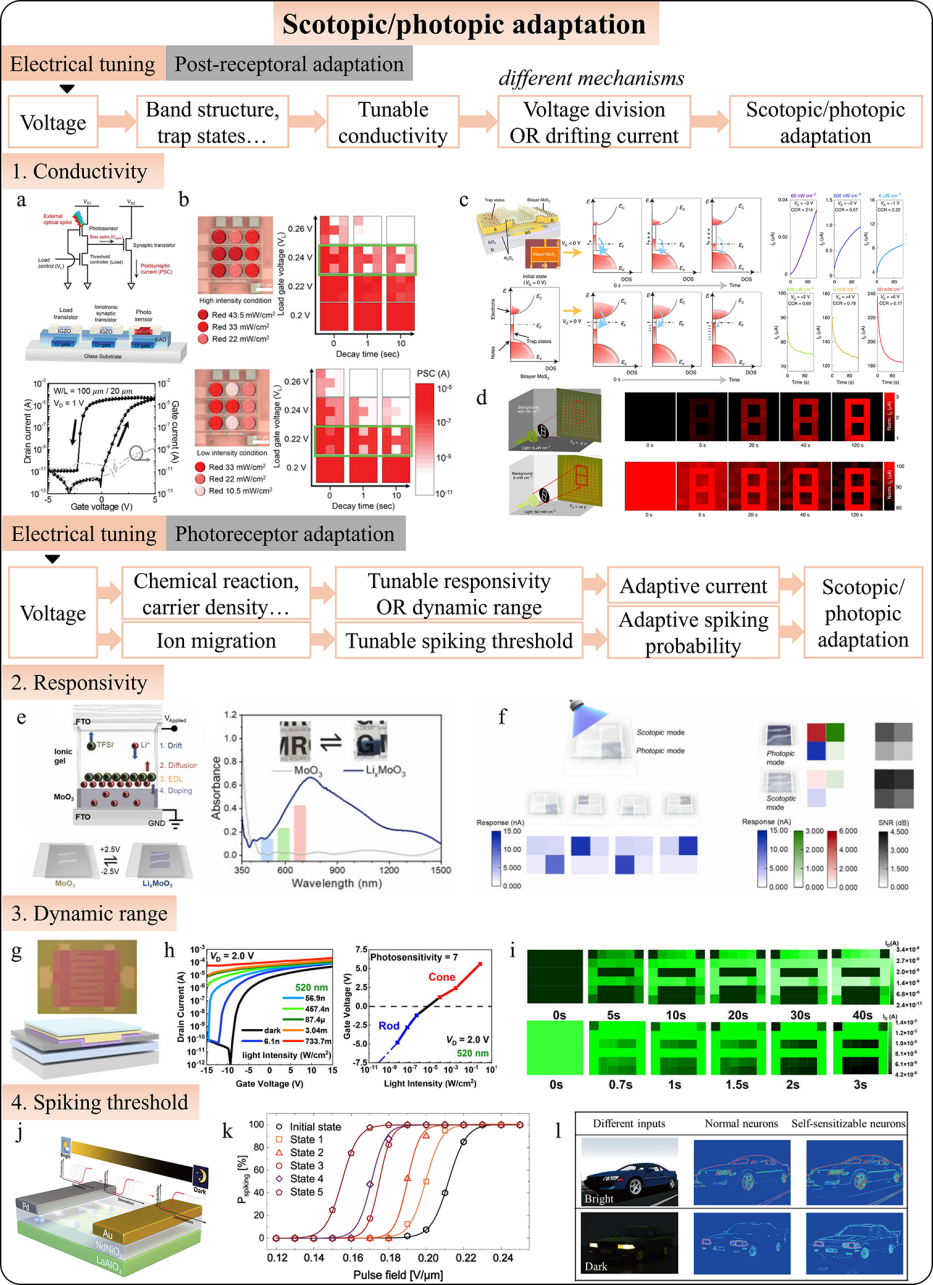
Electrically tuned intelligent photodetectors for scotopic/photopic adaptation. (a) Schematics and transfer characteristics of the ionotronic synaptic transistor. (b) Light-adaptive optoelectronic neuromorphic circuit array for artificial visual perception. Adapted with permission from ref 285. Copyright 2019 WILEY-VCH Verlag GmbH & Co. KGaA, Weinheim. (c) Structure- and time-dependent characteristics of the MoS_2_ phototransistor. (d) Demonstration of scotopic and photopic adaptation. Adapted with permission from ref 286. Copyright 2022 The Author(s), under exclusive license to Springer Nature Limited. (e) Schematics of the MoO_3_ electrochemical gating configuration and absorbance spectrum of both the MoO_3_ and Li_*x*_MoO_3_ films. (f) Color and contrast sensitivity adaptive sensing to light exposure by MoO_3_-based artificial eye. Adapted with permission from ref 287. Copyright 2022 Elsevier Ltd. (g) Schematic diagrams of the quasi-2D perovskite/IGZO phototransistor. (h) Transfer characteristics and *V*_g_ versus light intensity. (i) Demonstration of dark and light adaptation. Adapted with permission from ref 288. Copyright 2023 AIP Publishing. (j) Schematic of the hydrogen-doped perovskite nickelate device. (k) Spiking probabilities of different sensitized states. (l) Demonstration of edge detections. Adapted with permission from ref 289. Copyright 2024 Elsevier Inc.

In addition to compensating for the existing signal, scotopic/photopic adaptation can occur as early as signal generation. These devices typically modify the photodetector’s figures of merit, such as responsivity and dynamic range. Responsivity controls the amplitude of signal change, while the dynamic range prevents signal loss from current saturation. Traditional voltage-dependent responsivity adjustments, such as bias changes in photodiodes or phototransistors, do not mimic the gradual adaptation process, as they occur too quickly. To replicate slow adaptation, mechanisms such as electrochromic switching or ion migration are often used.

Mathews et al. demonstrated electrically tunable responsivity using inorganic electrochromic transistors.^287^ By applying an electrochemical stimulus, they transformed the MoO_3_ layer into Li_*x*_MoO_3_ (Figure 9e), enhancing absorption and enabling reversible switching between color-sensitive photonic vision and contrast-sensitive scotopic vision (Figure 9f). Dynamic range can also be tuned for contrast improvement under varying light conditions. Zhou et al. adjusted the dynamic range by varying gate–source voltage (*V*_GS_),^288^ emulating the transition between rod and cone cells in photoreceptors, similar to the retina’s negative feedback mechanism (Figure 9g–i). In recent works, tunable responsivity can also be achieved with optical lens with custom-made apertures, simulating variable pupil of cats.^295^ However, simulating the slow adaptation process could remain a challenge for these intrinsically fast works.

This strategy extends to spiking signals, as well. Wang et al. reported a phototransistor with a floating gate that tunes the spike threshold for illumination adaptation, similar to tunable responsivity but in spike form.^296^ Zhang et al. developed a self-sensitizing neuromorphic device based on ion migration (Figure 9j) that adjusts its activation function autonomously, enhancing sensitivity by shifting the spike threshold with increasing input signals (Figure 9k).^289^ This allows the device to detect weak signals in dark environments (Figure 9l), although it requires light to be encoded into spike signals first. The self-sensitizing characteristic, although rare, shows promise for developing devices with automatic adaptation capabilities, potentially simulating feedforward stimulation in the future.

While electrical tuning effectively simulates vision adaptations based on feedback mechanisms, it struggles to replicate feed-forward processes, which operate independently of higher-level systems. For automatic adaptation, intensity information should be processed directly within the sensor, bypassing the higher-level systems. Zhu et al. addressed this by developing a structure with two complementary bulk heterojunctions (BHJs) that enable automatic adaptation.^297^ The device’s optically tuned characteristics arise from the interaction between field-effect modulation and the photovoltaic effect (Figure 10a). Upon light exposure, photoexcitation occurs in both BHJs, generating a transient response similar to that of a phototransistor. Electron trapping in the dielectric layer dynamically shields the gating field, allowing self-modulated photoadaptation and preventing saturation during intense exposures. The device essentially replaces electrical tuning with photovoltage generated by the photovoltaic effect, though it is limited to photopic adaptation as the photovoltage remains unidirectional with increasing light intensity (Figure 10b). To achieve scotopic adaptation, initial holes could be introduced into the dielectric layer as a floating gate, providing an opposite voltage in darkness that reverses as the light intensity increases. Additionally, human eyes adapt their response speed based on the lighting conditions. Luo et al. developed an organic transistor that simulates automatic scotopic adaptation for both amplitude and response speed.^300^ This device uses traps as rechargeable charge reservoirs, which fill under bright light and gradually release charges in darker environments, enhancing the current signal. The adaptation speed varies with light intensity as low light conditions require more time to fill all traps. However, this device can only simulate scotopic adaptation; to simulate photopic adaptation, traps with opposite charge preferences would be needed to neutralize oversaturated currents under bright conditions.

**Figure 10:**
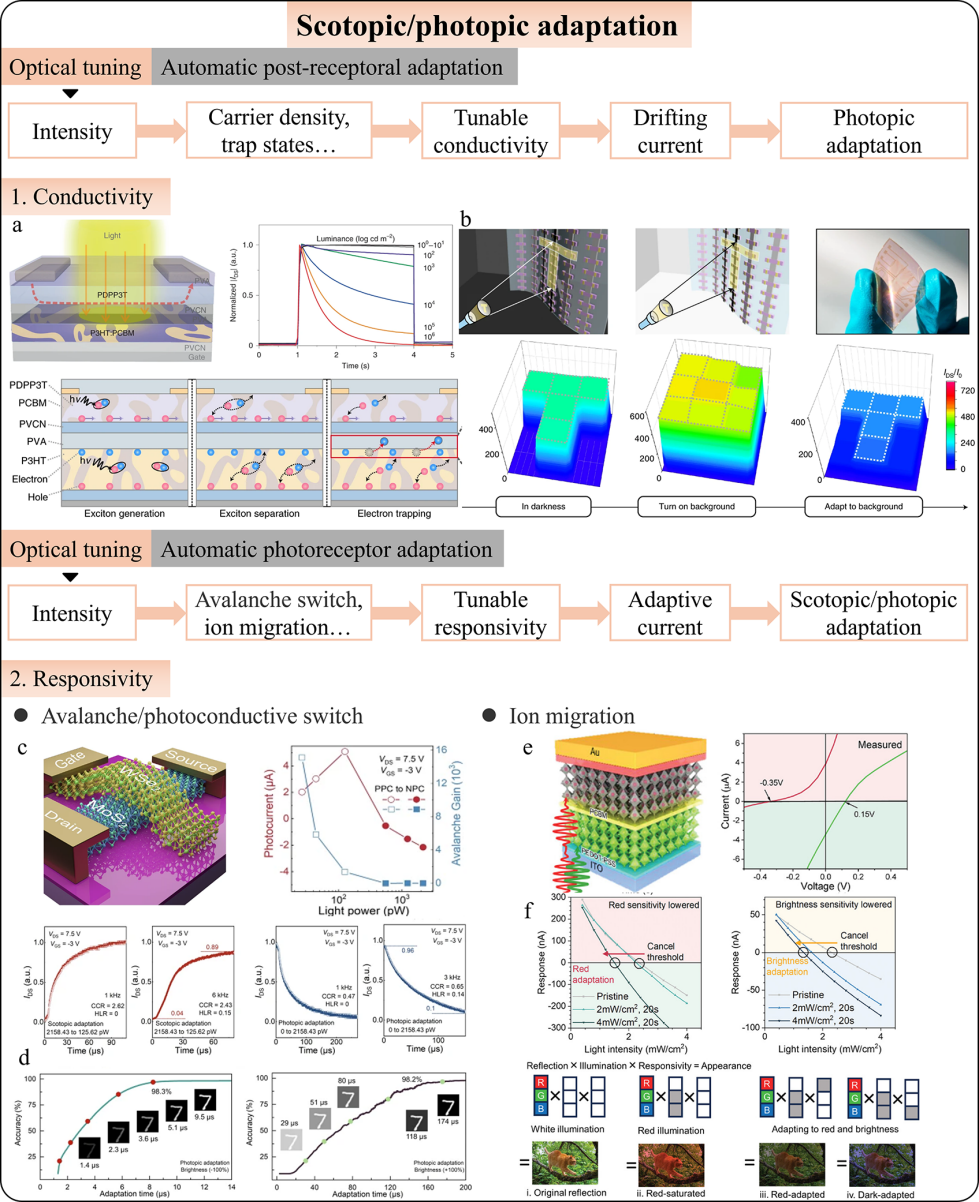
Optically tuned intelligent photodetectors for scotopic/photopic adaptation. (a) Schematics and working mechanism of OAAT with two complementary BHJs. (b) Demonstration of biomimetic visual perception. Adapted with permission from ref 297. Copyright 2021 The Author(s) under exclusive license to Springer Nature Limited. (c) Schematic diagram and light intensity-dependent avalanche of the device based on MoS_2_/WSe_2_ vdW heterostructure. (d) Recognition rate of adaptive machine vision as a function of time for scotopic and photopic adaptation. Adapted with permission from ref 298. Copyright 2024 The Author(s) under Creative Commons Attribution 4.0 International License (https://creativecommons.org/licenses/by/4.0/). (e) Device structure and response characteristics of the perovskite bipolar photodetector. (f) Demonstration of chromatic cancellation, adaptation, and partial color constancy. Adapted with permission from ref 299. Copyright 2024 Wiley-VCH GmbH.

In addition to compensating existing signals, the intrinsic response to light can be altered with current research focusing on optically tuned responsivity. This focus arises because adjusting variables such as dynamic range typically requires negative optical photoresponse to prevent current oversaturation in bright conditions, and current bidirectional optical modulation is usually instantaneous. Several methods now exist to achieve optically tuned variable responsivity. One strategy is to switch the operation regime between high and normal sensitivity modes. Li et al. reported a JFET device based on a MoS_2_/WSe_2_ vdW heterostructure that automatically switches between avalanche and photoconductive modes (Figure 10c).^298^ As light intensity increases, the built-in electric field triggering the avalanche effect is counteracted by the reversed photogenerated voltage at the MoS_2_/WSe_2_ junction, making avalanche gain inversely proportional to light power. This automatic adaptation improves the image quality in extreme lighting and enhances pattern recognition accuracy (Figure 10d).

Another approach uses ion migration to modify the absorbance and adjust the responsivity for specific wavelengths. Mathews et al. developed a perovskite bipolar photodetector with an opposite tandem structure,^299^ where a narrow bandgap perovskite is layered over a broad bandgap perovskite diode. The device exhibits a bipolar photoresponse to red and green light due to the series connection of diodes with opposite polarities (Figure 10e). Ion-mediated barrier modulation reduces the photoresponsivity to red after prolonged exposure, preventing red saturation in intense red-light environments. Similarly, dark adaptation is achieved in the Y_+_B_–_ channel as ion migration is mitigated under lower light intensity, enhancing responsivity (Figure 10f). This approach offers innovative ways to tune responsivity and could inspire further research in chromatic cancellation and wavelength-specific responsivity. In addition to works discussed above, phase separation in perovskite^301^ could be another optical tuning mechanism for automatic adaptation.

#### 4.2.3 Pattern Recognition and Neuromorphic Computing

The postmanufacturing tunability of photoresponsivity in intelligent photodetectors, like optoelectronic synapses, enables the hardware implementation of diverse neural network algorithms. In these systems, network weights are encoded as a photoresponsivity matrix, allowing for real-time multiplication with incoming images. Optoelectronic synapses with a single-layer perceptron (SLP) structure are already capable of handling basic pattern recognition tasks. Adding nonoptical hidden layers can significantly enhance their ability to perform more complex neuromorphic computing functions. Therefore, this section focuses specifically on pattern recognition only using tunable photodetectors, while briefly discussing potential methods for integrating nonoptical hidden layers. For a more in-depth examination of advanced neuromorphic computing architectures, we refer readers to other reviews.^41,302–305^

For pattern recognition and neuromorphic computing, light-induced tunability, such as retention time, important for tasks like denoising and motion detection, is less critical. Here, light primarily inputs image information, which can be replaced by encoded electrical signals if necessary. The key functionality lies in tunable responsivity, which represents neural network weights. By multiplying the light intensity with responsivity and summing the current, a matrix-vector product can be computed for one output neuron. To adjust for another neuron with different weights, the responsivity can be reprogrammed via a programming voltage, allowing for new weights to be set.

Choi et al. demonstrated a 2 × 2 single-layer perceptron for simple diagonal direction recognition using a mnemonic-opto-synaptic transistor (MOST) (Figure 11a),^306^ where different weights ensured only one neuron activated when the correct image was presented (Figure 11b). Mei et al. built a larger 64 × 64 array with an organic electrochemical transistor (OECT) for face recognition (Figure 11c).^307^ In this setup, light intensity mapped to image features influenced memory current, enabling the array to recognize faces based on their characteristic outlines (Figure 11d). Additionally, tunable photodetector arrays can process tactile signals, as demonstrated by Yang et al. with a visual-tactile multimodal recognition system using a two-terminal electrical synapse (Figure 11e).^308^ This design integrates feature extraction and multisensory fusion within a single reservoir, improving efficiency and accuracy by utilizing a mixed input rather than relying solely on electrical or optical signals (Figure 11f).

**Figure 11:**
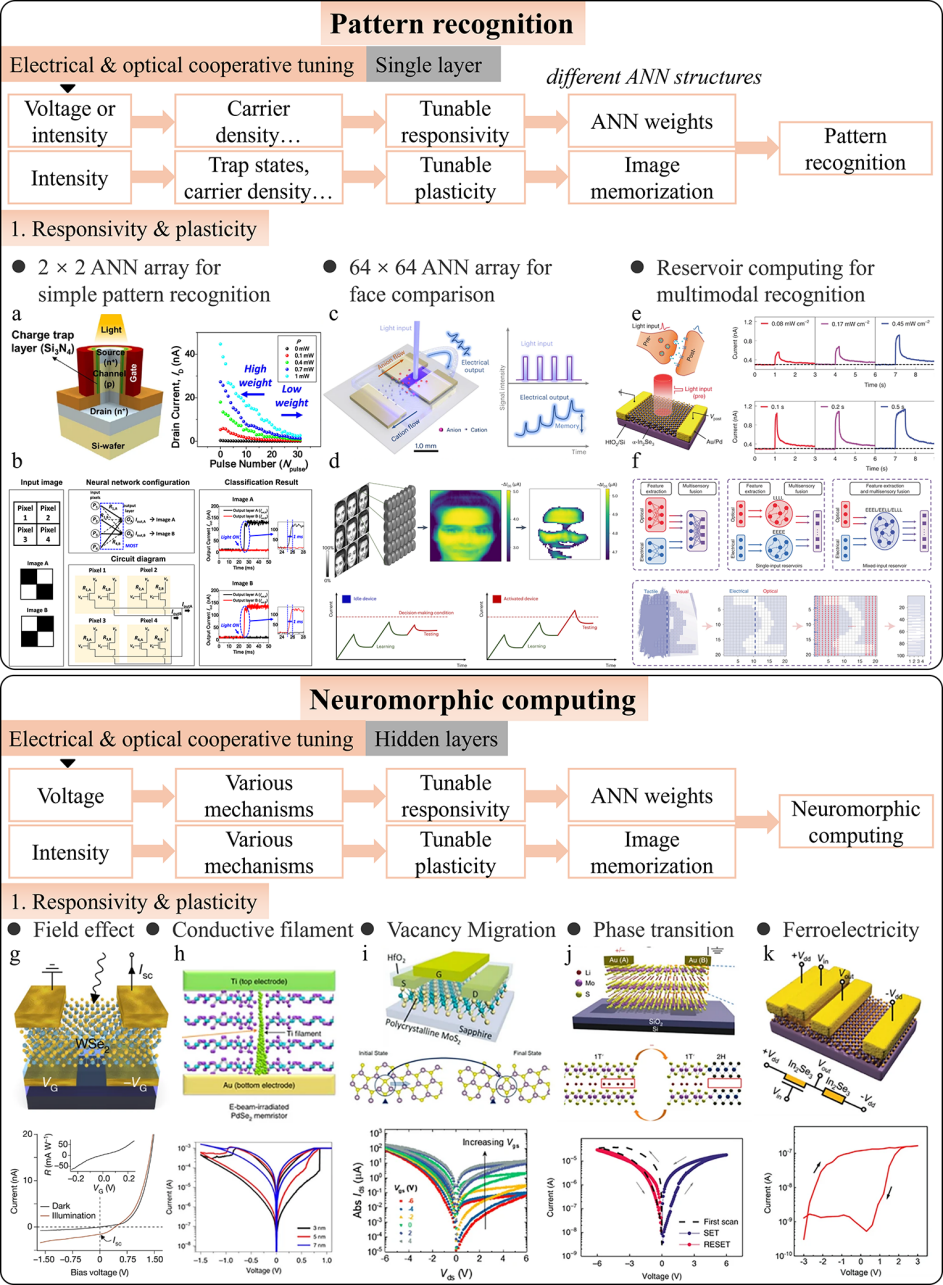
Intelligent photodetectors for pattern recognition and neuromorphic computing. (a) Schematics and depression behavior of the MOST. (b) Demonstration of pattern recognition. Adapted with permission from ref 306. Copyright 2022 The Author(s) under Creative Commons Attribution 4.0 International License (https://creativecommons.org/licenses/by/4.0/). (c) Schematic illustrations of optoelectronic synaptic OECT. (d) Demonstration of face recognition. Adapted with permission from ref 307. Copyright 2023 The Author(s) under exclusive license to Springer Nature Limited. (e) Schematic illustrations and intensity-dependent plasticity of the α-In_2_Se_3_ synapse. (f) Demonstration of multimode and multiscale reservoir computing. Adapted with permission from ref 308. Copyright 2022 The Author(s) under exclusive license to Springer Nature Limited. (g–k) Intelligent photodetectors for neuromorphic computing based on different mechanisms. (g) Field effect. Adapted with permission from ref 309. Copyright 2020 The Author(s) under exclusive license to Springer Nature Limited. (h) Conductive filament formation. Adapted with permission from ref 310. Copyright 2021 The Author(s) under exclusive license to Springer Nature Limited. (i) Vacancy migration. Adapted with permission from ref 311. Copyright 2019 WILEY-VCH Verlag GmbH & Co. KGaA, Weinheim. (j) Phase transition. Adapted with permission from ref 312. Copyright 2018 The Author(s) under exclusive license to Springer Nature Limited. (k) Ferroelectricity. Adapted with permission from ref 313. Copyright 2022 Wiley-VCH GmbH.

Tunable photodetector arrays can achieve some level of pattern recognition, but the limitations of a single perceptron layer require hidden layers not directly exposed to light. These hidden layers function as neuromorphic computing devices, an area witnesses significant advancement in recent years.^83–90,314–331^ A notable early example is the work by Mueller et al., who used a WSe_2_ photodiode array to build an artificial neural network (ANN).^309^ They added two gate electrodes to dynamically tune the n/p characteristics of WSe_2_, creating a lateral p–n junction with a tunable photoresponsivity (Figure 11g). However, because the modulation relies on the field effect, the network weights are lost once the voltage is removed. To create nonvolatile devices, mechanisms such as filament formation,^310^ defect migration,^311^ and phase^312^ or ferroelectric transitions^313^ can be employed (Figure 11h–k).

#### 4.2.4 Other Applications

Beyond the applications previously discussed, postmanufacturing tunability enables numerous other new functions. However, due to space constraints, we can only selectively highlight a few representative examples:

Das et al. developed a collision detector using a monolayer MoS_2_ photodetector combined with programmable FG nonvolatile memory (Figure 12a).^332^ The device can mimic the lobula giant movement detector (LGMD) escape response, where the current decreases without visual stimuli and shows a nonmonotonic trend when both visual excitation and programming inhibition are present (Figure 12b). The device can be programmed to detect different approach speeds by adjusting the back-gate bias. Based on similar ideas, collision detection can be realized on other material systems or structures.^296,335–337^

**Figure 12:**
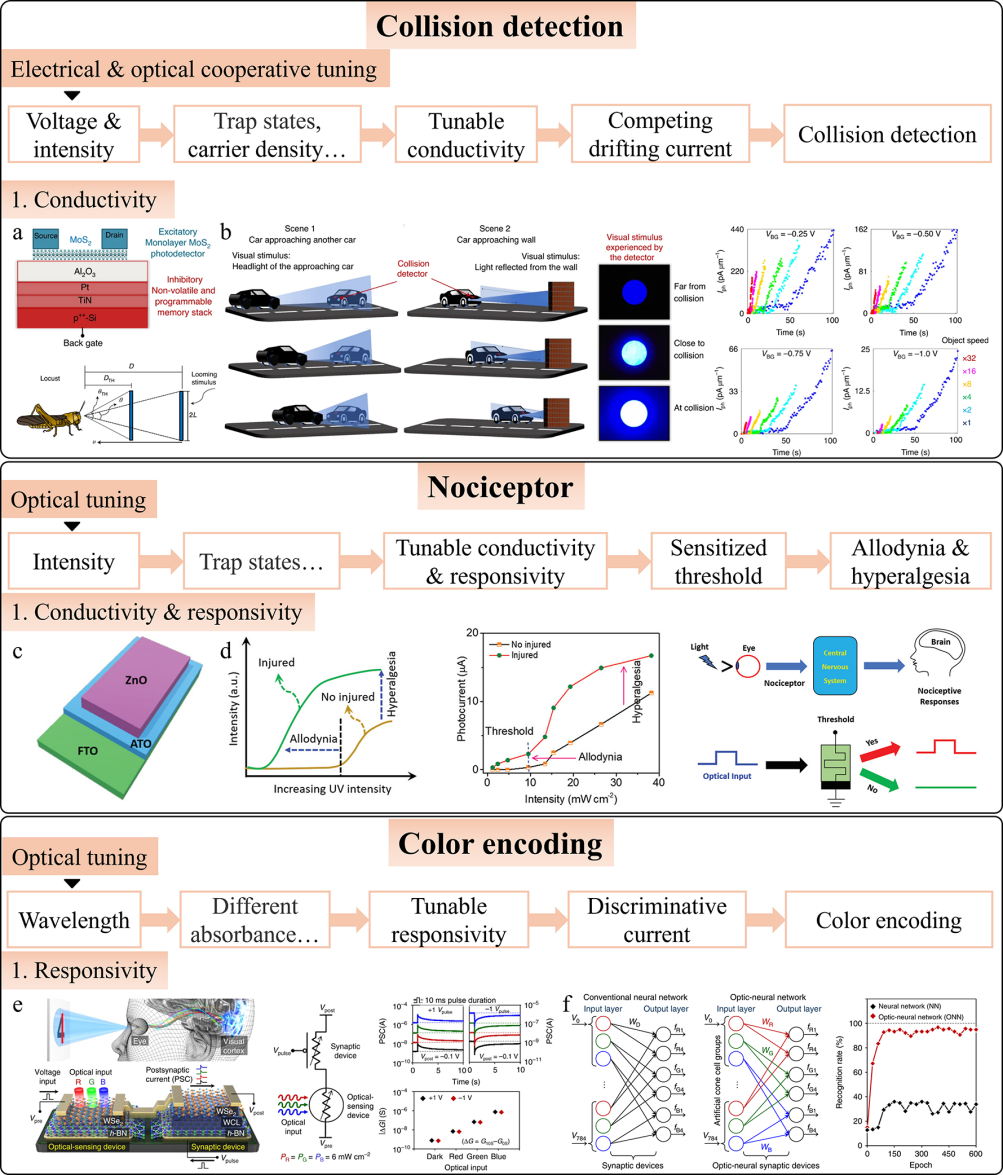
Intelligent photodetectors for collision detection, nociceptor, and color encoding. (a) Schematic of a biomimetic collision detector. (b) Schematics of collision detection. Adapted with permission from ref 332. Copyright 2020 The Author(s) under exclusive license to Springer Nature Limited. (c) Schematic of the device structure. (d) Nociceptive analogy between the human eyes. Adapted with permission from ref 333. Copyright 2019 WILEY-VCH Verlag GmbH & Co. KGaA, Weinheim. (e) Schematic of the human optic nerve system, the h-BN/WSe_2_ synaptic device integrated with h-BN/WSe_2_ photodetector, and the simplified electrical circuit for the ONS device. (f) Colored and color-mixed pattern recognition based on an artificial optic-neural network. Adapted with permission from ref 334. Copyright 2018 The Author(s) under Creative Commons Attribution 4.0 International License (https://creativecommons.org/licenses/by/4.0/).

Besides, Kim et al. created a memristor that replicates nociceptor behavior, which alerts the body to potential harm (Figure 12c).^333^ The device showed a low photocurrent under low UV intensity (LUV) but exhibited enhanced photocurrent after exposure to high UV intensity (HUV). After HUV exposure, the device became more sensitive, responding to even low UV intensities, mimicking the “allodynia” and “hyperalgesia” behaviors of an injured nociceptor (Figure 12d). In addition to ZnO, many other material and devices can exhibit similar nociceptive behavior, making nociceptor relatively a popular choice for optoelectronic synapses.^338–344^

Additionally, color encoding plays a crucial role in both artificial vision and image preprocessing. Seo et al. demonstrated an h-BN/WSe_2_ synaptic device with wavelength-tunable responsivity (can also be considered as tunable spectral response), enabling color recognition as a preprocessing step for image encoding.^334^ This device generates a discriminative current varying by several orders for different wavelengths, similar to how trichromatic cone photoreceptors in human eyes adapt (Figure 12e). This color encoding method has been shown to improve recognition rates, particularly for digits with mixed RGB colors, compared with conventional neural networks (Figure 12f). Building on the concept of discriminative current for different wavelengths, color encoding can be achieved using various other device structures and material systems.^345–347^ For instance, similar effect can be achieved by mixing different quantum dots together in a-IGZO phototransistor.^348^

### 4.3 Summary

This section reviews the advanced functions enabled by the tunable temporal response dynamics of intelligent photodetectors, particularly in terms of responsivity, conductivity, and synaptic plasticity. These functions, traditionally reliant on peripheral analogue circuits, can now be achieved through dynamic modulation within a single photodetector device—thereby reducing system complexity and reducing energy consumption.

Despite these promising advances, key limitations remain. The achievable modulation speed, long-term operational stability, and breadth of tunable parameters are often constrained. Moreover, integrating multiple tunable metrics within a single device can introduce trade-offs, where enhancing one function may unfavorably affect another. Scalability and reproducibility also pose challenges, especially for devices based on low-dimensional materials. Additionally, electrical tuning frequently necessitates complex control architectures, highlighting the need for strategies that enable autonomous optical adaptation. Lastly, most existing studies focus on steady-state tuning behavior, which limits the capacity to encode or extract temporal- or frequency-domain information. Future investigations may delve into time-related performance metrics, such as tunable frequency response and noise characteristics, to fully leverage the temporal processing potential of intelligent photodetectors.

## 5 Advanced Functions of Intelligent Photodetectors with Tunable Spectral Response

Spectral response is one of the key characteristics of photodetectors, as it describes how sensitive a photodetector is to the incident light of each wavelength. It also determines the working spectral range and, consequently, the specific applications of the photodetector. Conventional photodetectors typically exhibit a broadband photoresponse, lacking intrinsic spectral selectivity. In order to achieve color vision or other wavelength-specific applications, these photodetectors must be combined with additional optical filters or dispersive optics. However, these approaches complicate the system integration. Moreover, in conventional photodetectors, spectral sensitivity is generally not reconfigurable in run-time. This rigidity hinders the device from achieving advanced functions such as adapting to color-casting illumination conditions and color/spectrum perception. This section reviews recent advances in intelligent photodetectors featuring tunable or switchable spectral responses and explores their emerging applications.

### 5.1 Encrypted Optical Wireless Communications

Intelligent photodetectors can be engineered to selectively respond to specific wavelengths, enabling advanced wavelength-based encoding schemes for encryption. For instance, Fu et al. developed a dual-polarity photodetector with highly selective responsivities in visible and near-infrared (NIR) bands, as shown in Figure 13a.^349^ The device incorporated a visible-light-absorbing perovskite layer and a NIR-absorbing organic BHJ layer sandwiched between a pair of low-work function contacts, forming a back-to-back diode configuration (Figure 13b). The spectral response of the device could be altered by applying different bias voltages (Figure 13c), enabling it to switch between four distinct operational modes: “visible-only mode”, “NIR-only mode”, “addition mode”, and “subtraction mode”. The device exhibits an unprecedented fast switch speed exceeding 500 kHz between different operation modes (Figure 13d). The fast switching speed has been leveraged to establish a novel encryption method based on arithmetic relations, supporting data transmission at rates up to 200 Kbit s^–1^ in the encrypted optical communication links (Figure 13e).

**Figure 13:**
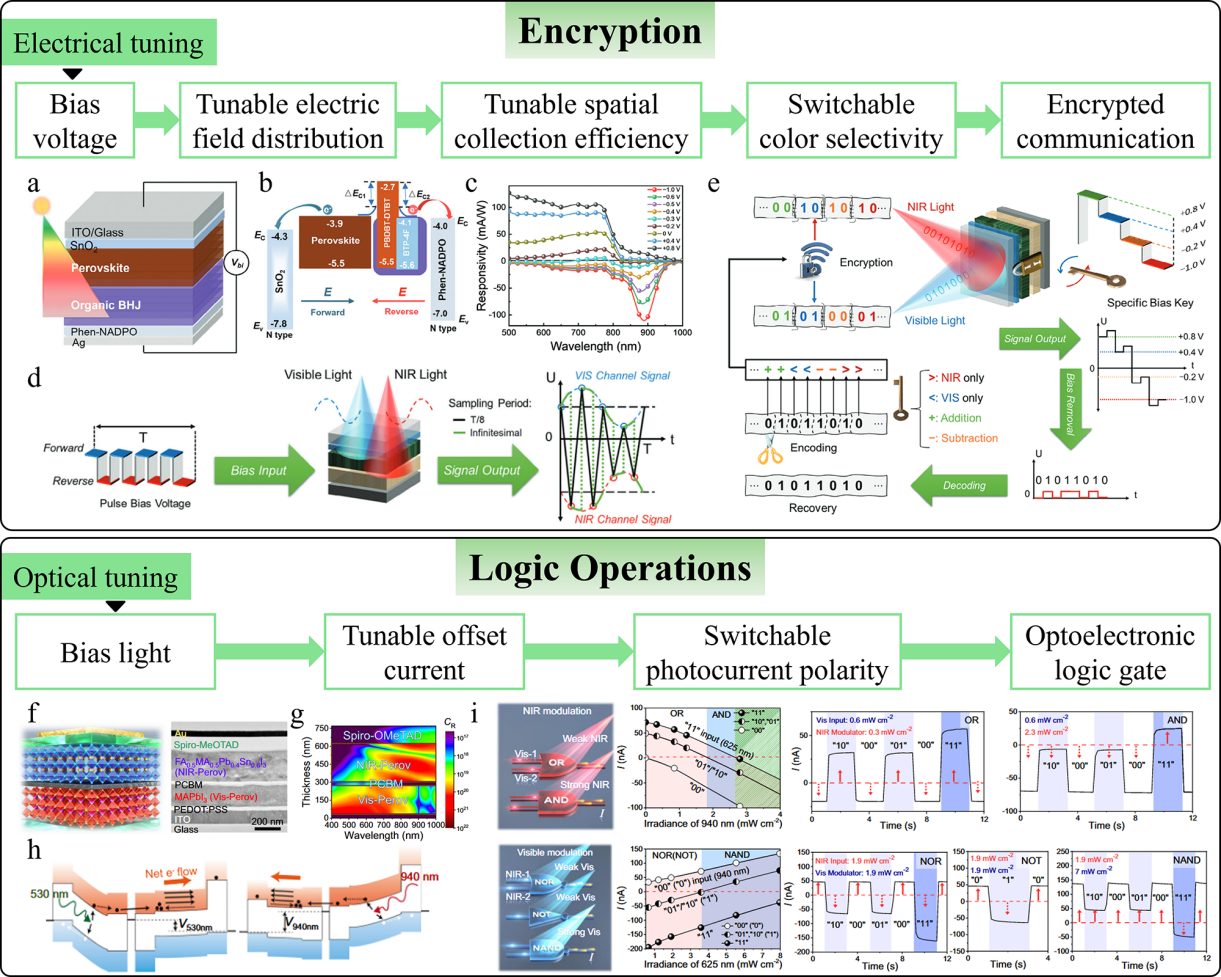
Intelligent photodetector with tunable spectral response for encrypted communication and logic operations. (a) Device structure, (b) band diagram, and (c) bias-dependent spectral response of the bias-switchable dual-polarity photodetector. (d) Schematic illustration of the bias-switching mode measuring the intensity of two wavelengths in turns. (e) Operational scheme of encrypted communication. Adapted with permission from ref 349. Copyright 2024 Wiley-VCH GmbH. (f) Device structure of vertically stacked dual-polarity perovskite photodetector. (g) Wavelength-dependent charge generation rate distribution. (h) Energy band diagrams under illumination of 530 and 940 nm light. (i) Demonstration of optoelectronic logic gates operation via a single photodetector. Adapted with permission from ref 356. Copyright 2022 The Author(s) under Creative Commons Attribution 4.0 International License (https://creativecommons.org/licenses/by/4.0/).

Bias-switchable dual-band photodetectors, fabricated using materials like organic semiconductors,^350–353^ perovskites,^95,354^ and hybrids,^355^ have successfully demonstrated secure wireless optical communication across the UV–vis–NIR spectral range, highlighting the versatility of this strategy. This approach also offers enhanced flexibility in communication protocols, making them highly adaptable for next-generation optical wireless systems.

### 5.2 Optoelectronic Logic Gate

Beyond bias tuning, dual-polarity photodetectors can also be programmed by bias light illumination, demonstrating their application as optoelectronic logic gates (OELGs). By definition, OELGs convert multiple optical inputs directly into digital signals based on fundamental logic operations (AND, OR, NOT, NAND, and NOR), facilitating high-speed optical data processing and communication.

In recent advancements, Pak et al. introduced a reconfigurable OELG using perovskite photodetectors with a back-to-back p^+^-i-n-p-p^+^ diode structure.^356^ This device utilized vertically stacked narrow-bandgap and wide-bandgap perovskites (Figure 13f). Due to the wavelength-dependent location of photocarrier generation, the device exhibited opposite polarity photocurrents under visible and NIR illumination (Figure 13h). To demonstrate the optoelectronic logic gate operations, 625 and 940 nm light were selected as either optical modulation or input signals, as shown in Figure 13i. The “AND” and “OR” gates responding to 625 nm signal light input was achieved with 940 nm bias light modulation. Similarly, under modulation of 625 nm light, the photodetector can demonstrate “NOR”, “NOT”, and “NAND” gates by responding to 940 nm input signals. The output is determined by the polarity of the photocurrent, which enhances the accuracy and reliability in the presence of current or electrical noise. A 64-device optoelectronic logic gate array has been demonstrated, showcasing potential for technology scaling-up.

OLEGs based on intelligent photodetectors with switchable color sensitivity have also been realized using various material systems, including BP/MoS_2_ heterostructure,^357^ CdTe/SnSe heterojunctions,^358^ GaN nanowires,^359,360^ demonstrating the versatility of this approach across different semiconductor platforms. Despite their advantages in speed and energy efficiency, OELGs face challenges that hinder their practical adoption. Key issues include the instability of materials and devices, difficulties in compact integration with electronic circuits and light sources, and limited compatibility with the CMOS fabrication processes. Overcoming these challenges through material innovation, novel device design, and advanced manufacturing techniques will be essential for their large-scale implementation. It is important to note that OELGs based on tunable spectral response only represent one branch of recent advances in this field; for a more comprehensive discussion, readers may refer to other focused reviews.^361,362^

### 5.3 Color Vision and Spectrum Reconstruction

#### 5.3.1 Color and Multiband Vision

Conventional color imaging with CCD or CMOS sensors relies on broad-band photodetectors integrated with color filter arrays. This integration requires precise alignment during fabrication, which increases the complexity and overall fabrication cost. Additionally, the use of a filter array can result in significant loss of incident light (e.g., 50–70% for Bayer filter array). One alternative approach is to vertically stack multiple photodetectors into a single pixel, as illustrated in Figure 1b.^25,27–29,363^ However, such configuration would typically contain multiple contacts inserted between devices, which requires a complicated etching process and therefore reduces the spatial fill factor. To address these limitations, researchers are exploring intelligent photodetectors with tunable color sensitivities that offer promising alternatives by enhancing efficiency, reducing light loss, and simplifying device architecture.

Jung et al. developed a two-terminal organic–inorganic hybrid perovskite photodetector with bias-modulated multicolor discrimination, as shown in Figure 14a.^364^ The device leverages the aforementioned back-to-back diode configuration to selectively utilize photocarriers from different active layers under positive or negative voltage (Figure 14b). By carefully tuning the bandgap and thickness of the absorbing layers, desired color discrimination can be achieved. Leveraging the flexibility in spectral response tailoring, Jung et al. introduced a dual-detector platform with complementary color sensitivities, which achieved color perception in the RGB color space (Figure 14c). The color sensor demonstrated exceptional accuracy in identifying arbitrary colors, offering a spatially efficient and cost-effective solution for color perception. Similar concept of full-color imaging has been demonstrated with organic photodetectors.^365^

**Figure 14:**
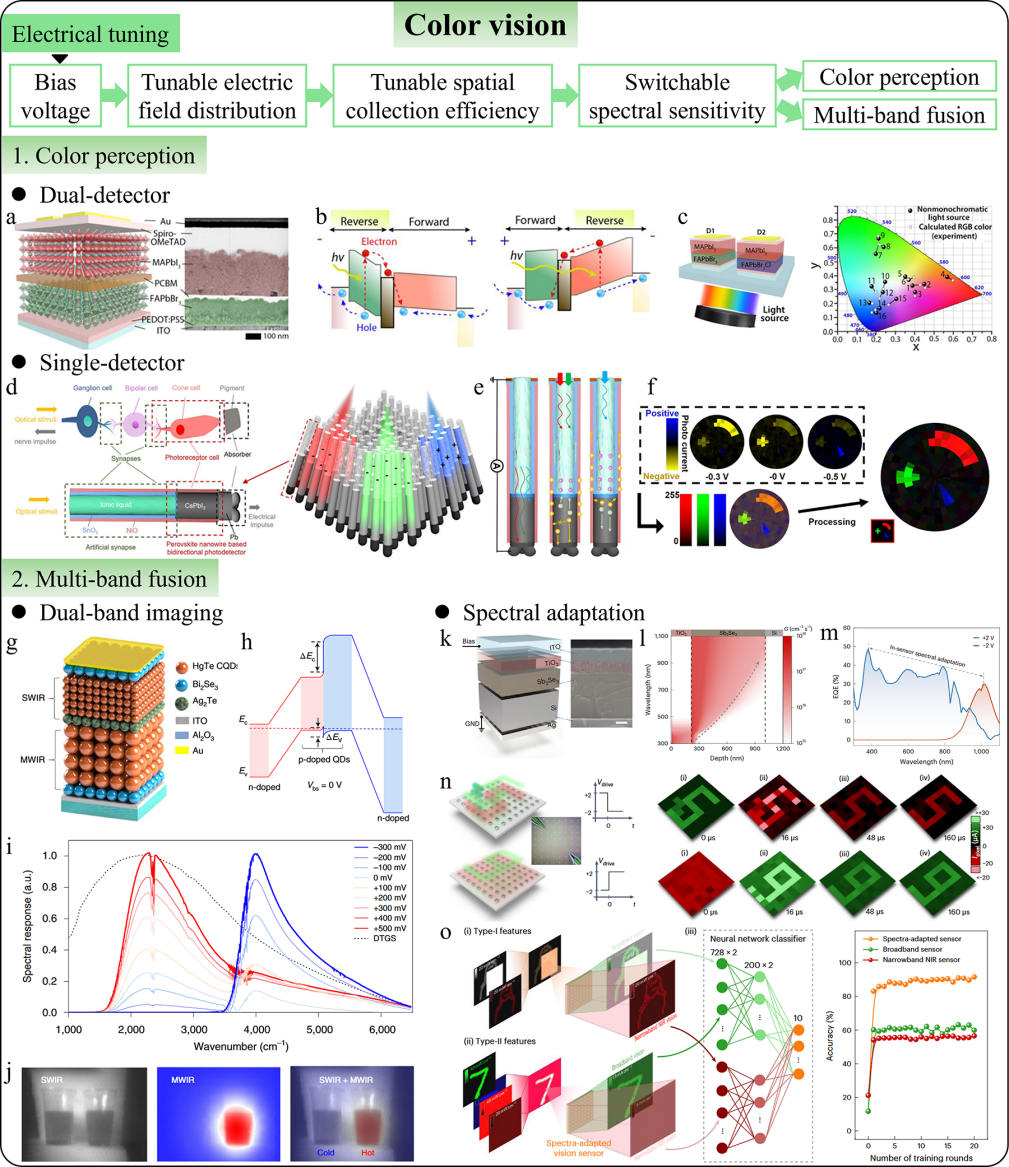
Intelligent photodetector with a tunable spectral response for color vision. (a) Schematic and SEM image of the perovskite filter-free photodetector. (b) Energy band diagram under forward and reverse bias conditions. (c) Dual-detector platform for color perception. Adapted with permission from ref 364. Copyright 2020 Wiley-VCH GmbH. (d) Schematics of retina neurons and a neuromorphic color sensor. (e) Wavelength-dependent photocarrier generation and charge carrier dynamics. (f) Demonstration of color pattern reconstruction. Adapted with permission from ref 366. Copyright 2023 The Author(s) under Creative Commons Attribution 4.0 International License (https://creativecommons.org/licenses/by/4.0/). (g) Schematic illustration, (h) energy diagram, and (i) bias-dependent spectral response of the dual-band colloidal quantum dot photodetector. (j) Demonstration of dual-band IR imaging. Adapted with permission from ref 367. Copyright 2019 The Author(s) under exclusive license to Springer Nature Limited. (k) Schematic and SEM image of the device structure. (l) Depth profile of charge generation rate. (m) Spectral response under positive or negative bias voltage. (n) Demonstration of spectral adaptation. (o) Operational scheme and recognition accuracy of the spectra-adapted vision system. Adapted with permission from ref 379. Copyright 2024 The Author(s) under exclusive license to Springer Nature Limited.

While the back-to-back configuration with dual active layers can effectively achieve bias polarity-determined color discrimination, it is inherently limited to two states and cannot realize color perception within a single device. To overcome this, Fan et al. introduced an innovative device using a SnO_2_/NiO double-shell nanotube filled with ionic liquid, placed atop a CsPbI_3_/NiO core–shell nanowire (Figure 14d).^366^ The perovskite nanowire was 1-μm-long so that the difference in penetration depth can be leveraged to generate a wavelength-dependent photocarrier distribution. For example, under shorter wavelength illuminations (e.g., blue light, 0 V bias), the carriers are mostly generated on the top of the nanowire and yield positive photocurrent (Figure 14e). On the contrary, the device generates negative photocurrent under longer wavelength (green and red light, 0 V bias) illuminations. However, in a single-step (single-bias voltage) measurement, red and green colors can hardly be separated (Figure 14e). To address this issue, a small negative bias can be applied, which reduces the height of the Schottky barrier at the CsPbBr_3_/Pb interface. Consequently, carriers generated by red light near the interface can flow to external circuit more easily and generate relatively stronger negative photocurrent than green light, reaching a better color discrimination. Eventually, a three-bias voltage measurement scheme is deployed for a better color recognition standard, as shown in Figure 14f. The novel photodetector was implemented in a hemispherical bionic retina and demonstrated excellent filter-free color vision.

The concept of color vision can be extended into multiband vision beyond the visible spectrum. For instance, Guyot-Sionnest et al. developed a dual-band infrared photodetector using stacked HgTe quantum dots of two different sizes, as shown in Figure 14g.^367^ Short-wave infrared (SWIR) and midwave infrared (MWIR) were absorbed by the separate quantum dots layers. Back-to-back diodes were formed by doping the QDs into n-i-p-i-n configuration (Figure 14h), and the same dual-polarity operational scheme as discussed before were applied to generate dual-band photoresponse (Figure 14i). Figure 14i demonstrated the fusion of the SWIR band and MWIR band. The SWIR image mapped the reflected light from the target illuminated by a tungsten lamp, showing texture and detail of the object. On the other hand, the MWIR image mapped the thermal emission from the target, providing the temperature distribution. This photodetector, covering two important atmospheric windows for infrared imaging, eliminates the need of multiple sensors and offers a cost-effective solution for sensor fusion. Based on the similar concept and device design, researchers have also developed photodetectors for VIS-NIR, NIR-MWIR or UV-IR dual-band imaging using Ge–Si heterojunctions,^368,369^ 2D vdW heterojunctions,^370–372^ perovskite,^373,374^ organic materials^211^ and hybrids.^375^

As one of the most important application scenarios, multiband fusion technologies can maintain the imaging quality under nonideal illumination conditions. Mismatch between the spectral response of image sensors and the spectrum of the surrounding environment can lead to low imaging quality in conventional CMOS-/CCD-based machine vision systems, thus leading to the ineffective extraction of visual features.^63,371,376–378^ Inspired by vision systems of pacific salmon, Chai et al. developed a spectrally adaptive vision sensor based on arrays of back-to-back photodiodes.^379^ As shown in Figure 14k, the device was composed of a stacked structure of ITO/n-TiO_2_/p-Sb_2_Se_3_/n-Si/Ag. Combining the sequentially narrowing bandgap formed by TiO_2_/Sb_2_Se_3_/Si and the self-filtering effect of the 800 nm-thich Sb_2_Se_3_ layer, wavelength-dependent photocarrier generation depth was achieved, as depicted in Figure 14l. Consequently, the device exhibited either a broadband visible or narrowband NIR response under positive or negative external bias, respectively (Figure 14m). The device can quickly adapt between the two modes, greatly enhancing the contrast in imaging (Figure 14n). As shown in Figure 14o, the adaptation ability allowed improved accuracy in pattern recognition under nonideal illumination conditions, such as color-casting and background-glare interference.

#### 5.3.2 Spectrum Reconstruction

Color sensors and multiband sensors based on intelligent photodetectors can deliver intensity information from several wavelength bands. However, a wide range of applications including materials characterization, medical diagnostics, food science and biosensing requires ultrafine spectral details across hundreds of narrow bands, which inevitably requires using spectrometers.^380^ Conventional benchtop spectrometers achieve high resolution over a broad spectral range by utilizing dispersive components, long optical paths, and intricate moving mechanisms. However, this leads to bulky sizes and high costs, limiting the device integration with portable and wearable platforms.

To address the need for compact, cost-effective spectrometers and hyperspectral imaging systems, there has been a concentrated effort toward the miniaturization of these devices. There are two main approaches used in the miniaturization of spectrometers. The first follows the same principle as conventional spectrometers, where monochromatic light is fed into photodetectors using dispersive optics, narrowband filters, or Fourier-transform-based systems. This strategy offers good performance, but optical-path-length restrictions prevent reducing the device size to the submillimeter scale. The second leverages computational reconstructive algorithms with arrayed broadband photodetectors that are designed to exhibit distinct spectral responses, such as nanowires with spatial composition gradients,^33,40^ structurally colored silicon nanowire arrays^35^ or filter-encoded photodetector arrays.^31,36^ These methods do not require narrow bandpass optics and can be scaled down to submillimeter footprints. However, the strategy of photodetector arrays are restricted by the need for beam uniformity, precision chemical synthesis, and nanofabrication, as well as trade-off between footprint and resolution.

Recent advancements in intelligent photodetectors with tunable spectral response have led to success in replacing photodetector arrays with single photodetectors for spectrum characterization. Central to the single-device operational scheme is the achievement of a distinct spectral response under different modulation conditions. For instance, Xia et al. introduced a novel scheme for mid-infrared spectroscopy using a single tunable black phosphorus photodetector, within an active area footprint of only 9 × 16 μm^2^, as shown in Figure 15a.^51^ This device utilized the Stark effect to achieve a tunable photon absorption in the black phosphorus channel. Consequently, the device, working in intrinsic photoconduction mode, can deliver tunable spectral response, as shown in Figure 15b. The spectral information was sampled by measuring the photocurrent under a number of biasing displacement fields (Figure 15c) and then computationally reconstructed (Figure 15d). This work represents a simplified and cost-effective method for mid-infrared spectroscopy and spectral imaging.

**Figure 15:**
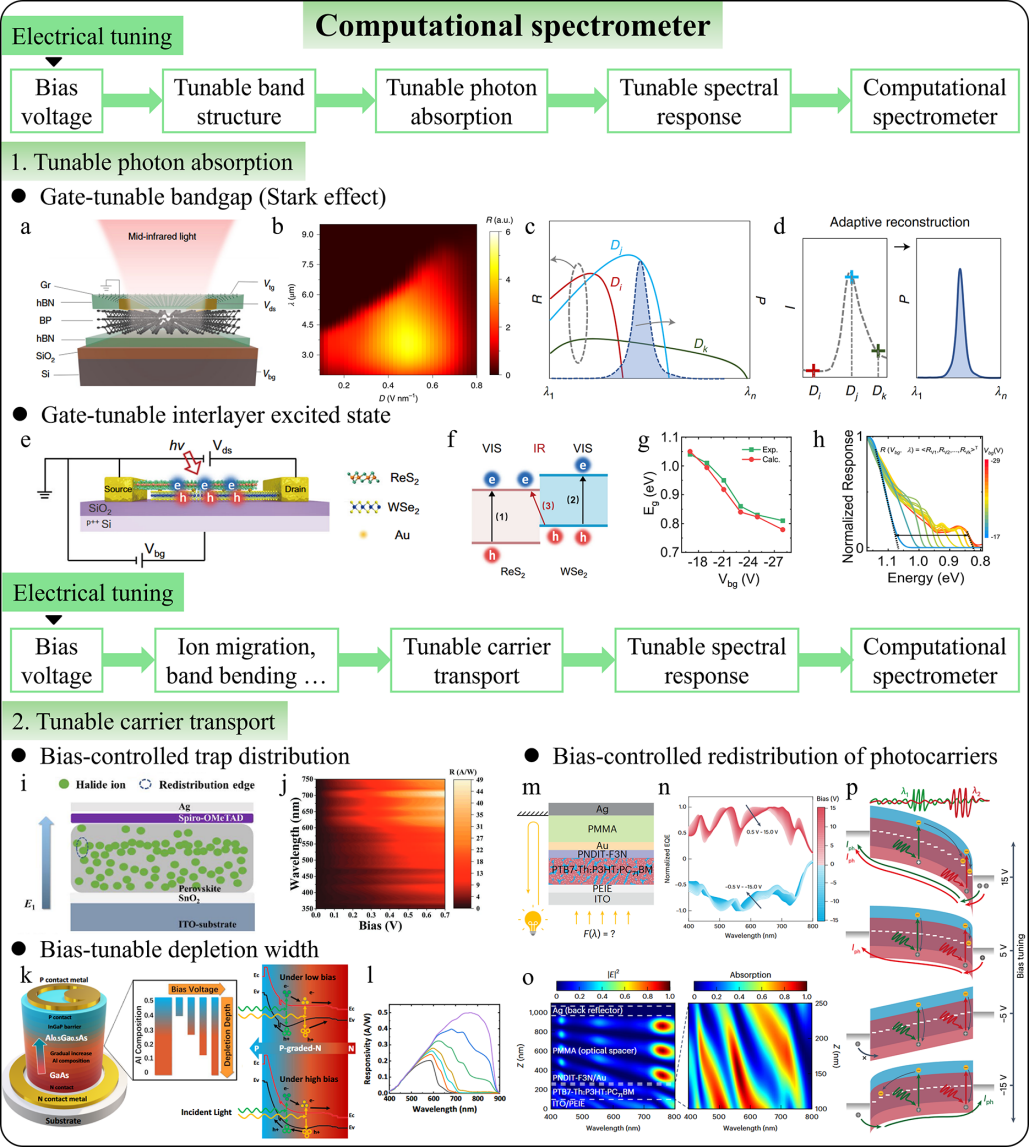
Miniaturized spectrometers enabled by intelligent photodetectors with tunable spectral response. (a) Schematic and (b) spectral response as a function of biasing displacement field of the black phosphorus transistor. (c) Sampling and (d) reconstruction process. Adapted with permission from ref 51. Copyright 2021, The Author(s), under exclusive license to Springer Nature Limited. (e) Schematics of the 2D-vdW heterojunction spectrometer. (f) Photoexcited transition path in the heterojunction, including intralayer and interlayer transition. (g) Gate-tunable band offset. (h) Gate-tunable spectral response. Adapted with permission from ref 381. Copyright 2022 The Author(s) under Creative Commons Attribution 4.0 International License (https://creativecommons.org/licenses/by/4.0/). (i) Schematic illustration of the device structure and ion migration in the perovskite spectrometer. (j) Bias-tunable spectral response. Adapted with permission from ref 52. Copyright 2022 Wiley-VCH GmbH. (k) Schematic illustration and (l) bias-tunable spectral response of the p-graded n junction spectrometer. Adapted with permission from ref 386. Copyright 2024 The Author(s) under Creative Commons Attribution 4.0 International License (https://creativecommons.org/licenses/by/4.0/). (m) Schematic illustration and (n) bias-tunable spectral response of the optical spacer-integrated organic spectrometer. (o) Optical field distribution and (p) carrier dynamics in the device. Adapted with permission from ref 387. Copyright 2024 The Author(s) under exclusive license to Springer Nature Limited.

While the Stark effect provides a straightforward pathway to achieve tunable spectral response, the spectral range is limited by the intrinsic properties of the materials. An alternative approach leverages bias-tunable interlayer excited states in 2D van der Waals heterostructures. Zhang et al. reported a miniaturized spectrometer based on a ReS_2_/Au/WSe_2_ heterostructure, as shown in Figure 15e.^381^ By intercalating heavy metal atoms (Au) at the interface, the device exhibited an enhanced interlayer transition dipole moment. Consequently, the heterostructure exhibited profound sub-bandgap absorption, which was contributed by the interlayer excited states (Figure 15f). Moreover, the energy level offset between ReS_2_ and WSe_2_ can be effectively modified by the gate voltage (Figure 15g), delivering a tunable spectral response (Figure 15h). Exploiting the gate-tunable photoresponse and a ridge regression algorithm, an ultraminiaturized NIR spectrometer was realized with a footprint of only 6-μm, offering an attractive solution for on-chip infrared spectroscopy.

The richness of modulation mechanisms in 2D materials and heterojunctions has made them extensively studied for single-device spectrum reconstruction. Besides the Stark effect and tunable interlayer excited states, other effects such as the quantum-confined Franz–Keldysh and Burstein–Moss effects have also been employed to modulate the bandgap and light absorption in a black phosphorus/MoS_2_ heterojunction.^382^ In another example, Yoon et al. demonstrated 2D van der Waals heterojunction spectrometer enabled by bias-tunable interlayer transport in MoS_2_/WSe_2_ transistor^383^ and black phosphorus/MoS_2_.^384^ Du et al.^385^ exploited the giant electrostriction effect in a semifloating MoS_2_ homojunction to tune the bandgap and carrier kinetics, modulating both the amplitude and relaxation time. Using a dual-signal response and a deep neural network algorithm, this device achieved spectrum reconstruction with a resolution of 1.2 nm and a waveband number of 380. These diverse approaches enhance spectral response features, broaden the operational wavelength regime, and offer improved accuracy and versatility for spectral detection.

Beyond tunable photocarrier generation, postgeneration processes can also be utilized to manipulate spectral responses for spectrum sampling. For instance, Li et al. developed a single dot perovskite spectrometer leveraging the tunable photoconductive gain induced by ion migration (Figure 15i).^52^ Ion migration is generally considered as a challenge faced by perovskite materials and needs to be eliminated, as it is not favorable for long-term operational stability. However, in this work, Li^+^ ions were strategically introduced as additives to replace the migration of intrinsic halogen ions and acted as a “regulator” for the alteration of the ion distribution state. The ion migration resulted in redistribution of carrier traps (Figure 15b), which, in turn, manipulated photoconductive gain in a wavelength-dependent manner. Consequently, the perovskite photodetector exhibited the desired bias-tunable spectral response, as shown in Figure 15j. The in situ modulation strategy overcomes the footprint-resolution restriction and potentially can be fabricated into arrays for hyperspectral imaging.

Carrier transport manipulation offers other possibilities for generating tunable spectral responses. For instance, Wang et al. developed a single-pixel-photodetector spectrometer based on the AlGaAs/GaAs p-graded-n junction.^386^ Unlike the gradient material nanowires that work in a lateral fashion,^33^ the p-graded-n spectrometer relies on a stacking longitudinal compositionally graded epitaxial structure to achieve wavelength-dependent photocarrier distribution (Figure 15k). At low reverse bias, holes generated by longer wavelength incident light absorbed in the low-Al side of the Al_*x*_Ga_1–x_As layer are blocked by the valence band barrier from the high-Al side and InGaP layer. Thus, these holes cannot diffuse into the depleted region and thus have no contribution to photocurrent (Figure 15k). When the reverse bias increases, the active layer is further depleted, allowing for the photogenerated carriers by the longer wavelength light to contribute to the photocurrent. Consequently, the device exhibited a longer cutoff wavelength as the bias voltage increases (Figure 15l). Leveraging the bias-tunable spectral response and reconstruction algorithm, high accuracy and robustness in spectrum reconstruction was achieved. The fabrication of this device is scalable and compatible with the standard III–V process, making it suitable for hyperspectral imaging.

Similarly, the bias-controlled redistribution of photocarriers in organic photodetectors can also manipulate the spectral response. Zhao et al. reported a microsized spectrometer based on a photomultiplication-type organic photodetector (PM-OPD), as shown in Figure 15m.^387^ A trilayer contact consisting of a transparent back contact, an optical spacer, and a back reflector was developed to increase the optical path length difference between the incident and reflected light. Consequently, the wavelength dispersion is substantially enhanced throughout the region of photocarrier generation (Figure 15o). In PM-OPDs, only the photocarriers trapped near the contact contribute to the photocurrent. As for the photocarriers far away from the contact, it can only be utilized after being driven toward the interface by a sufficiently high bias voltage (Figure 15p). Such spatially constrained utilization and bias-controlled redistribution of photocarriers, together with the use of the integrated optical spacer, collectively result in the bias-tunable spectral response (Figure 15n). The solution processability of the organic materials ensures the scalability and performance uniformity, allowing for scaling up the device fabrication and producing spectrometer arrays for spatial-scanning-free hyperspectral imaging.

### 5.4 Summary

In this section, we explore advanced functionalities enabled by the tunable spectral responses of intelligent photodetectors, such as encrypted communication, logic operations, color vision, multiband fusion, and computational spectroscopy. Traditionally, these functions required auxiliary optical or electronic components; however, performance modulation in intelligent photodetectors now allows for their integration into single devices.

Despite these advances, several challenges persist. First, the prevalent use of back-to-back diode configurations facilitates straightforward bipolar responses but restricts switching to only two or a few states. Exploring sophisticated carrier manipulation mechanisms and integrating functional optical structures are necessary to achieve more versatile spectral tuning. Second, addressing operational stability, compatibility with CMOS fabrication processes, and seamless integration with other electronic and optical components is crucial. Third, in pursuit of detecting more information, the community is now exploring high-dimensional sensors capable of characterizing the spectrum, polarization, phase as well as spatial and temporal information.^41,388–391^ It is still very challenging to achieve high-dimensional photodetection within a single or even a few intelligent photodetectors. Overcoming these obstacles through material innovation, novel device design, and advanced manufacturing techniques is essential for widespread implementation and the expansion of new applications for intelligent photodetectors with tunable spectral responses.

## 6 Switchable Functions of Intelligent Photodetectors

The postmanufacturing tunability of intelligent photodetectors extends beyond tuning some specific figures of merit. These devices can also be dynamically reconfigured to operate in distinct modes, thereby providing switchable functionalities. In this section, we review recent advances that enable intelligent photodetectors to switch between conventional photodetection and other functions. Specifically, Section 6.1 focuses on multifunctional light emitting and detecting devices; Section 6.2 discusses devices capable of switching between high-speed photodetection and neuromorphic sensing; and Section 6.3 highlights systems that achieve multisensory perception. By consolidation of multiple functions into a single device, intelligent photodetectors simplify system design and integration, opening new avenues for compact and adaptive optoelectronic applications.

### 6.1 Multifunctional Light Emitting and Detecting Devices

The p-n (or p-i-n) junction is the core of many optoelectronic devices such as photodiodes, solar cells, and light-emitting diodes (LEDs). Light emission (from LEDs) and light absorption (by detectors and solar cells) are reciprocity processes. Upon charge injection under the forward bias in LEDs, electrons and holes recombine radiatively, generating a light emission. Upon light excitation in detectors or solar cells, photogenerated carriers are separated under the built-in or externally applied electrical field and then collected by electrodes. Given that these devices all share the same architecture, it is possible to create multifunctional light-emitting and -detecting devices by simply adjusting the bias conditions.

Bao et al.^13^ demonstrated a dual-functional perovskite diode that can both emit and detect light by modulating the bias between forward and reverse conditions (Figure 16a). The switchable functionality enables bidirectional optical signal transmission between either two identical chips or two identical devices on a single chip. Notably, the authors developed a monolithically integrated photoplethysmogram (PPG) sensor for vital sign monitoring. In this configuration, a pair of identical diodes was fabricated on a single chip: one diode operates under forward bias to emit light, while the other, under reverse bias, detects the light reflected from the skin vasculature (Figure 16b). This setup enabled the effective tracking of arterial pulse waves. Furthermore, in an optical communication system, a pair of identical chips were used, with each chip capable of switching between transmitter and receiver modes via bias adjustment, thereby establishing a bidirectional data link (Figure 16c). This work underscores the versatility and potential of dual-functional perovskite diodes for a wide range of applications, from healthcare monitoring to optical communication.

**Figure 16:**
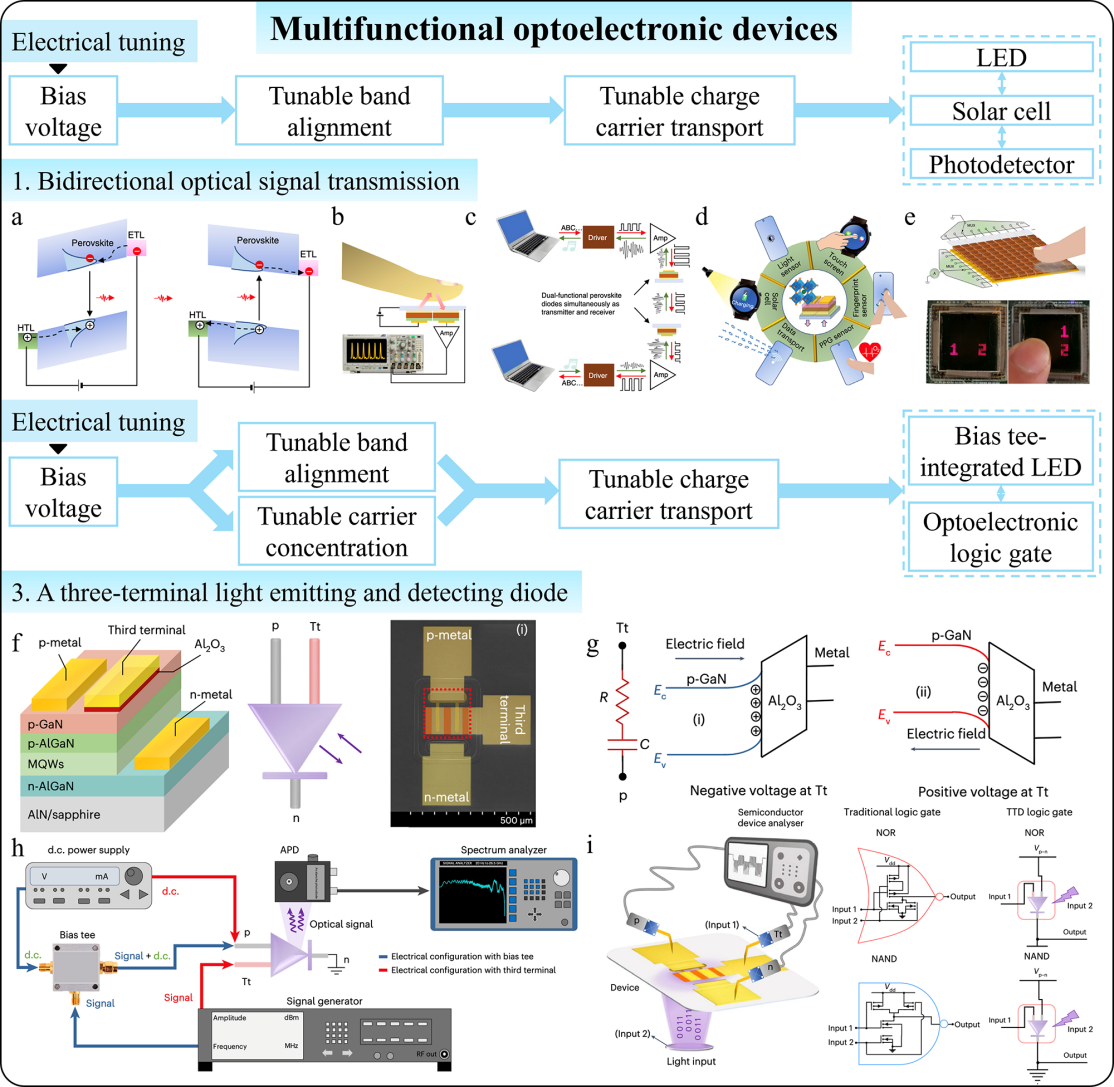
Multifunctional optoelectronic devices enabled by intelligent photodetectors. (a) Schematics of the energy diagram of the perovskite diode under forward bias as an LED (left) and reverse bias as a photodetector (right). (b) Demonstration of the PPG sensor for arterial pulse wave tracking using the dual-functional diode. (c) Schematics of using the dual-functional diode as the transmitter and receiver in a bidirectional communication system. Adapted with permission from ref 13. Copyright 2020, The Author(s), under exclusive license to Springer Nature Limited. (d) Illustration of the multifunctional display screen. (e) Demonstration of “inputting information function” of the display. Adapted with permission from ref 116. Copyright 2024 The Author(s) under Creative Commons Attribution 4.0 International License (https://creativecommons.org/licenses/by/4.0/). (f) Device structure of the three-terminal diode. (g) Schematic band diagrams of the Tt under negative and positive voltage conditions. (h–i) Illustration of utilizing the three-terminal diode as a transmitter with integrated bias tee and optoelectronic logic gate. Adapted with permission from ref 392. Copyright 2024 The Author(s) under exclusive license to Springer Nature Limited.

As a prominent application of LEDs, display screens are extensively used as user interfaces in consumer electronics. By incorporating additional functions with LED displays, favorable features like ultrathin, lightweight, and large screen-to-body ratios can be achieved (Figure 16d). Building on their previous research, Gao et al. recently demonstrated a multifunctional LED display, as shown in Figure 16f.^116^ Operating under reverse bias as photodetectors, the display enabled touch sensing by mapping the photocurrent of each pixel. This touch sensing capability further allowed for interactive displays with input functions, as shown in Figure 16e. Additionally, the display has been demonstrated to function as a fingerprint sensor, ambient light sensor, PPG sensor, data receiver, and solar cell (power supply). This work underscored the advantages of multifunctional intelligent photodetectors in consumer electronics applications.

Beyond two-terminal intelligent photodetector structures, Memon et al. introduced a third terminal into the conventional diode structure, as shown in Figure 16f.^392^ This third terminal, consisting of metal/Al_2_O_3_ deposited on the p-layer, provided an additional degree of freedom for controlling charge carrier transport through field-effect modulation of carrier concentrations, as depicted in Figure 16g. When functioning as an LED, the intensity could be adjusted via a third terminal. Thus, the third terminal served as an integrated bias tee, offering excellent modulation bandwidth for optical communication (Figure 16h). In its role as a photodetector, the third terminal regulated the photocurrent. By using both incident light intensity and the voltage applied to the third terminal as input signals, the authors demonstrated reconfigurable optoelectronic logic gates (Figure 16i). The device architecture demonstrated in this work represented a promising route toward future compact integrated optoelectronic systems.

### 6.2 Dual-Mode Operation: High-Speed Photodetection and Neuromorphic Sensing

Intelligent photodetectors capable of switching between high-speed photodetection and neuromorphic sensing have also been developed. For instance, Thomas et al. demonstrated such dual-mode operation with a perovskite quantum dots-sensitized graphene FET, as shown in Figure 17a.^186^ The phototransistor exhibited fast photodetection when the gate voltage was set to 0 V, as shown in Figure 17b. However, when a positive gate voltage was applied, photogenerated electrons were trapped at trap centers within the graphene. This trapping hindered the recombination of photocarriers, allowing holes to continue flowing through the channel under the influence of the drain voltage, resulting in persistent photoconduction (Figure 17c). The tunable synaptic behavior of this device was leveraged in a facial recognition system, providing integrated capabilities for optical information detection, processing, and retention.

**Figure 17:**
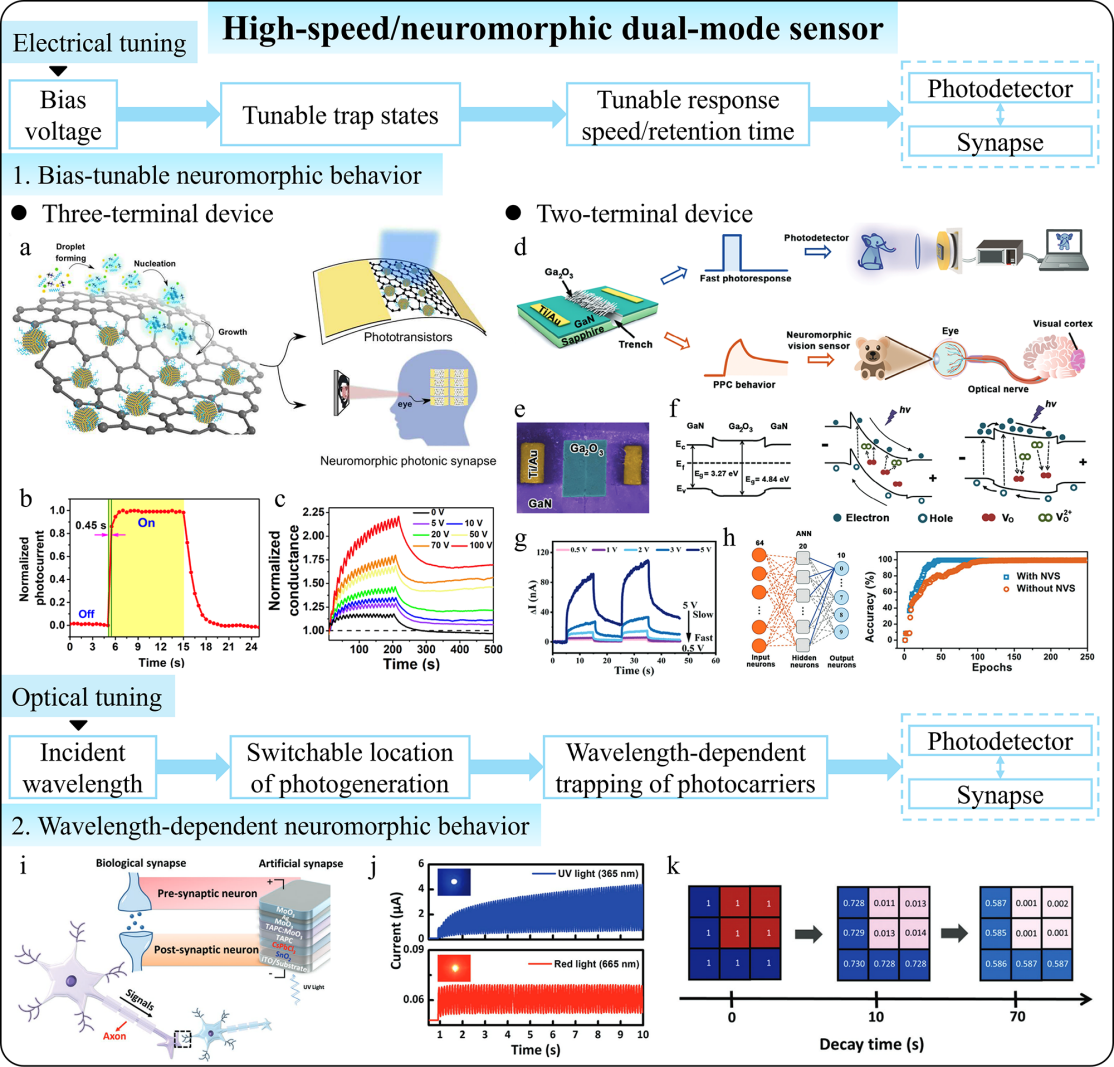
Intelligent photodetectors enabling switching between a high-speed photodetector and neuromorphic sensor. (a) Schematics of the perovskite quantum dots-sensitized graphene FET. (b) Fast photoresponse under 0 V gate voltage. (c) Synaptic behavior under positive gate voltage. Adapted with permission from ref 186. Copyright 2020, The American Association for the Advancement of Science. (d) Schematics, (e) SEM image, and (f) band diagrams of the dual-mode photodetector. (g) Bias-dependent response speed and synaptic behavior. (h) Schematics and accuracy of pattern recognition. Adapted with permission from ref 185. Copyright 2023 Wiley-VCH GmbH. (i) Schematics of the photodetector-synapse dual-mode device. (j) Wavelength-dependent synaptic behavior. (k) Demonstration of color-specific image memory. Adapted with permission from ref 393. Copyright 2020 Wiley-VCH GmbH.

The simultaneous integration of photodetector and neuromorphic sensor functionalities within a single device has also been achieved in two-terminal devices, which simplifies fabrication and lowers power consumption. Feng et al. demonstrated such a dual-mode device using a GaN/Ga_2_O_3_/GaN trench-bridged heterostructure, as shown in Figure 17d–e.^185^ The switching between modes was facilitated by the bias-controlled ionization of oxygen vacancies in Ga_2_O_3_, which acted as carrier traps and induced persistent photoconductance, as depicted in Figure 17f–g. The device’s fast photoresponse under low bias voltage was exploited for optical communication, while its synaptic behavior under high bias voltage was used to enhance pattern recognition accuracy and reduce power consumption (Figure 17h).

Beyond electrical modulation, dual-mode operation can also be achieved through optical tuning. Lin et al. demonstrated wavelength-dependent switching between photodetector and synapse functions in a device with the structure of ITO/SnO_2_/CsPbCl_3_/TAPC/TAPC:MoO_3_/MoO_3_/Ag/MoO_3_ (Figure 17i).^393^ UV light was absorbed by the CsPbCl_3_ layer, where photocarriers were trapped at the SnO_2_/CsPbCl_3_ interface, thereby triggering synaptic behavior under UV illumination. In contrast, red light was absorbed by charge-transfer states between TAPC and MoO_3_, which did not induce photocarrier trapping, resulting in a fast photoresponse (Figure 17j). This wavelength-switchable photodetector/synapse functionality was demonstrated in color-specific image memory (Figure 17k), suggesting new possibilities for mimicking the human visual and memory system.

Beyond simply detecting the light intensity information, Wu et al. demonstrated the integration of a spectroscopic sensor and a neuromorphic sensor within a single device.^394^ This was achieved using a SnS_2_/ReSe_2_ van der Waals heterostructure (Figure 18a). When the drain voltage was set to 0 V, photocarriers were unable to circulate within the channel, resulting in a fast photoresponse (Figure 18b). Upon application of a drain voltage, synaptic behavior and persistent photoconductance became prominent (Figure 18c). By applying a positive gate voltage, trapped carriers could be cleared, effectively erasing the photoconductance. Gate-tunable spectral response (Figure 18d) was achieved by regulating carrier transport, enabling the spectroscopic functionality. Thus, this device achieved a compact footprint of 19 μm, a bandwidth spanning from 400 to 800 nm, a spectral resolution of 5 nm, and a long-term image memory exceeding 10^4^ seconds, offering a promising alternative to traditional von Neumann architectures (Figure 18e–f).

**Figure 18:**
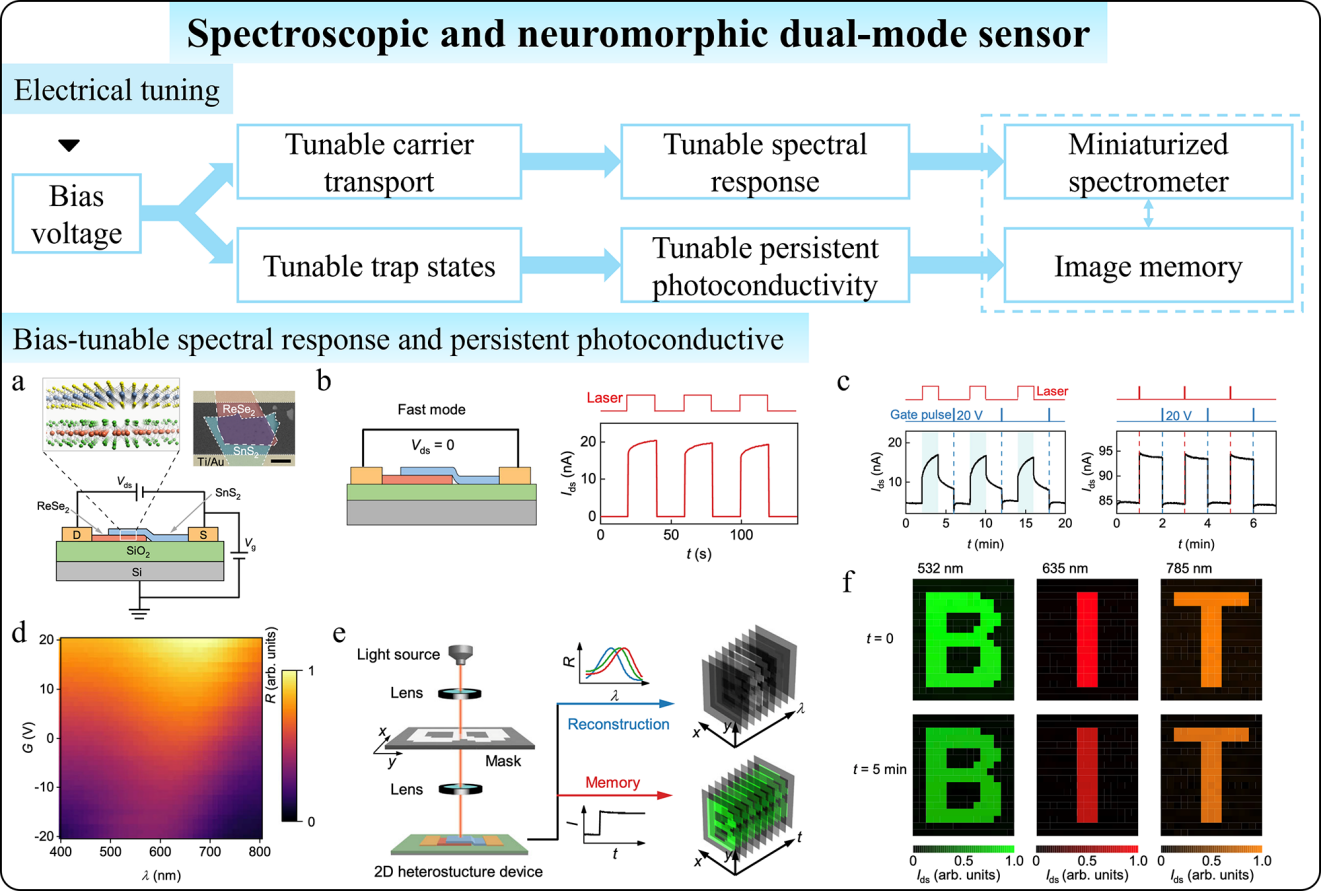
Miniaturized spectrometer with intrinsic long-term image memory. (a) Schematics of the SnS_2_/ReSe_2_ van der Waals heterostructure. (b) Transient photoresponse recorded under 0 V drain voltage. (c) Synaptic behavior and persistent photoconductance under 1 V drain voltage. (d) Gate-tunable spectral response. (e) Schematics of the dual-functionality as spectrometer and imaging memory. (f) Demonstration of image memory. Adapted with permission from ref 394. Copyright 2024 The Author(s) under Creative Commons Attribution 4.0 International License (https://creativecommons.org/licenses/by/4.0/).

### 6.3 Multisensory Perception

Intelligent photodetectors tuned by parameters beyond traditional electrical or optical methods have enabled multisensory perception within a single device. For instance, Jiang et al. developed a visual-chemical synapse using a monolayer of oxidized MXene (Figure 19a).^395^ This device was capable of simultaneously capturing visual (photon) and respiratory (hydroxyl) signals. The trapping of photocarriers produced postsynaptic photocurrents, while interactions between hydroxyl groups and oxidized vacancies led to the detrapping of the photocarriers (Figure 19b). As a result, the device exhibited a humidity-dependent synaptic behavior, as demonstrated in Figure 19c. By integrating visual and chemical sensing within a single device, the researchers were able to obtain neural excitability signals across six representative activities (Figure 19d). This capability showcases the device’s potential as a multimodal linking center for humanoid brain emulation.

**Figure 19:**
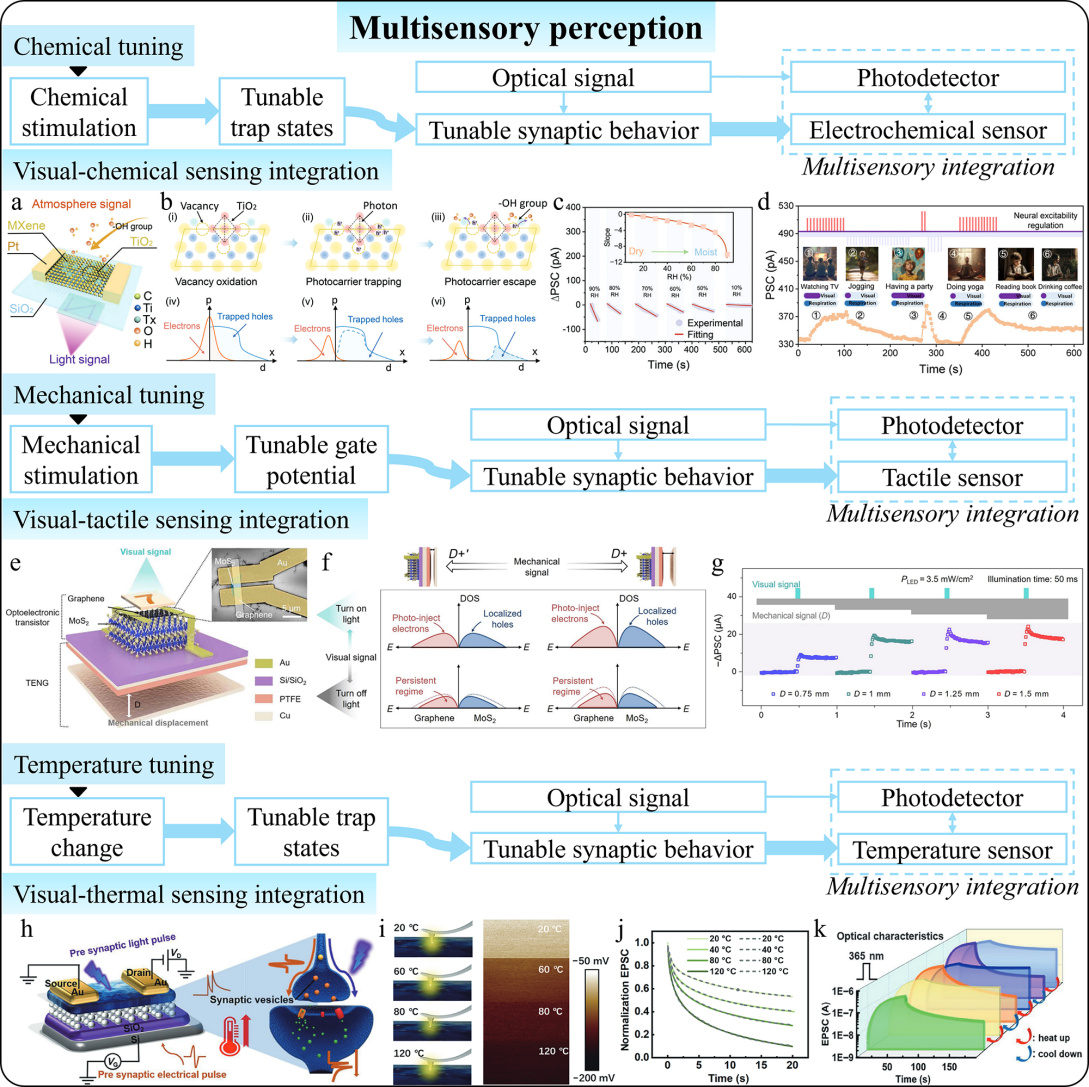
Intelligent photodetector enabling multisensory integration. (a) Schematics of a Mxene synapse. (b) Mechanism of light and hydroxyl interaction with Mxene. (c) Humidity-dependent synaptic behavior. (d) Demonstration of neural excitability signal acquisition based on visual-chemical sensing integration. Adapted with permission from ref 395. Copyright 2024 Wiley-VCH GmbH. (e) Schematic illustrations of a tactile/visual sensory system. (f) Schematic illustrations of the operational mechanisms. (g) Synaptic behavior under different mechanical modulation. Adapted with permission from ref 396. Copyright 2021 The American Association for the Advancement of Science. (h) Schematics of the artificial synapse with CsPbBr_3_/TiO_2_ as a floating gate layer. (i) Surface potential of CsPbBr_3_/TiO_2_ under different temperature. (j–k) Temperature-dependent synaptic behavior. Adapted with permission from ref 397. Copyright 2023 Tsinghua University Press.

Intelligent photodetectors with tactile and temperature sensing capabilities have also been demonstrated. Wang et al. developed an artificial synapse based on graphene/MoS_2_ heterostructure and an integrated triboelectric nanogenerator (Figure 19e).^396^ By controlling the charge transfer in the heterostructure with a triboelectric potential (Figure 19f), the optoelectronic synaptic behaviors can be readily modulated (Figure 19g). Consequently, the artificial synapse was capable of implementing mixed-sensory signals of visual and tactile input, emulating complex biological nervous systems. In addition, Guo et al. developed a temperature-controlled multisensory neuromorphic device based on floating-gate phototransistors (Figure 19h).^397^ CsPbBr_3_/TiO_2_ core–shell nanocrystals functioned as a floating gate, generating photocarriers upon illumination. The temperature-sensitive charge carrier trapping/detrapping in TiO_2_, characterized by surface potential (Figure 19i) resulted in tunable synaptic behavior (Figure 19j). Based on this relationship, the multisensory perception of temperature and optical signals was realized.

### 6.4 Summary

In this section, we reviewed recent advances in the switchable functions of intelligent photodetectors, which demonstrate how postmanufacturing tunability enables these devices to be dynamically reconfigured for a wide range of tasks. These innovations not only simplify system integration but also pave the way for compact and adaptive optoelectronic applications.

Despite these advances, several challenges remain. First, switching functions often involve trade-offs between performance metrics. For example, devices optimized for light harvesting may require a thicker active layer than those designed for light emission. Comprehensive studies are needed to elucidate the underlying mechanisms and identify the optimal balance. Second, switching between sensing distinct physical parameters and simultaneously sensing them represents two different levels of multisensory functionality. More advanced multisensory devices or systems should be capable of processing mixed signal inputs effectively. Third, stability and compatibility with existing electronics remain major concerns for new materials employed in these devices. Future research should focus on developing strategies to address these challenges and unlock the full potential of the intelligent photodetectors.

## 7 Conclusions and Outlook

In the discussions above, we highlighted the superior performance of intelligent photodetectors over static ones due to their adaptability. We have also shown how functions traditionally managed by the front-end optical components, separate detectors, emitters, and back-end circuits can now be integrated into photodetectors with tunable temporal response dynamics, spectral response, and functionality. Table 1 summarizes the progress of these advanced functions, which are categorized by tunable metrics. Development varies significantly across these metrics. Parameters such as conductivity and responsivity (whether used directly or for subsequent analogy calculations) have been thoroughly studied, with a wide range of applications in image preprocessing and artificial vision. In contrast, parameters like tunable response speed remain underexplored, despite their potential in bandwidth-related applications, such as spike neural networks or frequency-sensitive systems, highlighting a significant opportunity for further research. Moreover, electrical tuning currently dominates the field, but nonelectrical tuning methods also play a significant role in intelligent photodetectors. For example, optical tuning holds substantial promise for real-time automatic adaptation, while biochemical tuning could facilitate innovative biomedical applications by enabling responsive behaviors to biochemical signals. Consequently, advancing nonelectrical tuning methods could be directions worth future research efforts. Additionally, two-dimensional materials have been extensively studied and are arguably the most popular choice in related research. However, some of their performance metrics, particularly in areas such as dynamic range—impacted by complex carrier mechanisms—remain largely unexplored. Investigating alternative materials to overcome these limitations while leveraging their unique properties could expand the range of tunable metrics, thereby introducing new functionalities and enhancing performance. For example, perovskites could be utilized for ion migration and X-ray detection, while organic bulk heterojunctions may offer advantages in carrier trapping, bipolar behavior, and biosensing capabilities. Furthermore, certain applications benefit from device structures optimized for specific tunable parameters. For example, photoconductors and memristors are well-suited for plasticity, photodiodes excel in spectral response, and phototransistors are effective for responsivity and conductivity. Therefore, developing novel structures that integrate multiple tunable parameters simultaneously could open up space for new applications. Hybrid configurations, such as combinations of phototransistors with photodiodes or light-emitting diodes, could facilitate multimodal operations and enhance overall functionality. Finally, optimizing the tuning range and response time for specific parameters could further advance current applications. For instance, achieving broader photoresponsivity may improve algorithm weight resolution, while faster tuning could facilitate drift current-based compensation.

**Table 1:** Recent Progress of Intelligent Photodetectors Based on Various Tunable Figures of Merit^a^

Tunable parameters	Tuning methods	Materials	Structure	Advanced functions	Research progress
Dark current and photocurrent	Electrical	2DM,^76,238–240,286^ QD,^80^ MOF,^292^ nanocluster,^293^ inorganic,^285^ perovskite,^81^ ionic...^79^	PC & memristor,^81^ PD,^238^ PT^76,79,80,239,240,285,286,292,293^	Magnitude-related image preprocessing, vision adaptation, collision detection...	★★
	Optical	2DM,^101,102,245,332^ inorganic,^103,125,333^ organic,^246,297^ perovskite...^337^	PC & memristor,^125,245,333,337^ PD,^103^ PT^101,102,246,297,332^	Image memorization, automatic vision adaptation, collision detection, optoelectronic logic gate, nociceptor...	★★
Responsivity	Electrical	2DM,^63,65,66,68,71,260,264–267^ inorganic,^64,84,287,308^ organic,^67,70,85,263^ perovskite...^69,262^	PC & memristor,^65,66,71^ PD,^69,259,261,266^ PT^63,67,68,70,260,265,267,287,308^	Weight-related image preprocessing, vision adaptation, neuromorphic computing...	★★★
	Optical	2DM,^298,334^ QD,^348^ inorganic,^81,91,244,338^ organic,^307^ perovskite...^87,299^	PC & memristor,^82,87,338^ PD,^299^ PT^91,244,298,307,334^	Automatic vision adaptation, all-optical modulated neuromorphic computing, nociceptor, color encoding...	★★
Bandwidth	Electrical & optical	2DM,^266,267,294,385^ QD,^186^ nanocrystal...^300^	PD,^266^ PT^186,267,294,300,385^	Spike & speed-related image preprocessing, dual-signal spectrum reconstruction, PD/synapse switch...	★
Dynamic range	Electrical & optical	Inorganic,^288,398^ perovskite...^288,399^	PD,^398,399^ PT^288^	Range-related image preprocessing, vision adaptation...	★
Spectral response	Electrical	2DM,^370,371,381,383–385^ QD,^367^ inorganic,^368,379,386^ organic,^365,387^ perovskite...^95,97,364,366,373,374^	PC & memristor,^366,373^ PD,^95,365,367,370,371,374,379,384,386,387^ PT^97,381,383,385^	Spectral-related image preprocessing, encryption, color vision, spectrum reconstruction...	★★
Operation regime	Electrical, mechanical,^118–121,126,396^ ascoutic,^117,400^ thermal,^118,119,124,397^ chemical...^122,127,128,395^	2DM,^394–396^ QD,^186^ inorganic,^124,392^ organic,^122,127,397^ perovskite,^13,116^ TENG...^121,129,396^	PC & memristor,^129,395^ PD,^13,116,392^ PT^121,122,124,127,186,394,396,397^	Multimodal functions (photodetector/light emitting diode/optoelectronic synapse/solar cell...), multisensory perception (visual, tactile, auditory, olfactory, gustatory)...	★★
Plasticity	Electrical & optical	2DM,^74,242,268,273,274^ QD,^73,275^ inorganic,^58,61,272^ organic,^59,276^ perovskite,^62,75,243^ ionic,^72^ biological...^249^	PC & memristor,^58,61,72–75,242,249,272^ PD,^62,275^ PT^59,250,268,273,274^	Time-related image preprocessing, artificial vision involving memorization and adaptation...	★★★

a 2DM stands for two-dimensional materials; QD for quantum dots, MOF for metal–organic framework; TENG for triboelectric nanogenerator; PC for photoconductor; PD for photodiode; PT for phototransistor. To balance the number of references under each category in the table, only a selection of representative studies was included for certain categories.

In conclusion, intelligent photodetectors represent a transformative advancement in optoelectronics—not only by improving performance metrics, but also by fundamentally redefining the role of the photodetector itself. Enabled by postmanufacturing tunability across both spectral and temporal domains, these transcend passive light detection to actively perform sensing, preprocessing, and computing within the same physical unit. This evolution defines a new design paradigm that bridges materials science, photonics, electronics, and AI hardware, constituting the foundation of an emerging interdisciplinary research direction. Positioned at the nexus of adaptability and functionality, intelligent photodetectors are poised to become enabling technologies for future innovations in autonomous systems, neuromorphic computing, high-dimensional sensing, AI, and beyond.

Looking ahead, the advancement of intelligent photodetectors is likely to follow a path of increased tunability across a range of performance metrics, as shown in Figure 20. Establishing a universal platform for tunable metrics in optoelectronics, similar to field-programmable gate arrays (FPGAs) in electronics, would enable programmability for diverse functions. Incorporating tunable high-dimensional parameters like polarization and phase could unlock new applications, such as polarimeters, while integrating switchable nonoptoelectronic functions would allow these devices to perform more complex tasks. Additionally, simultaneous tuning of multiple parameters could break down existing functional barriers and create new, more sophisticated capabilities.

**Figure 20:**
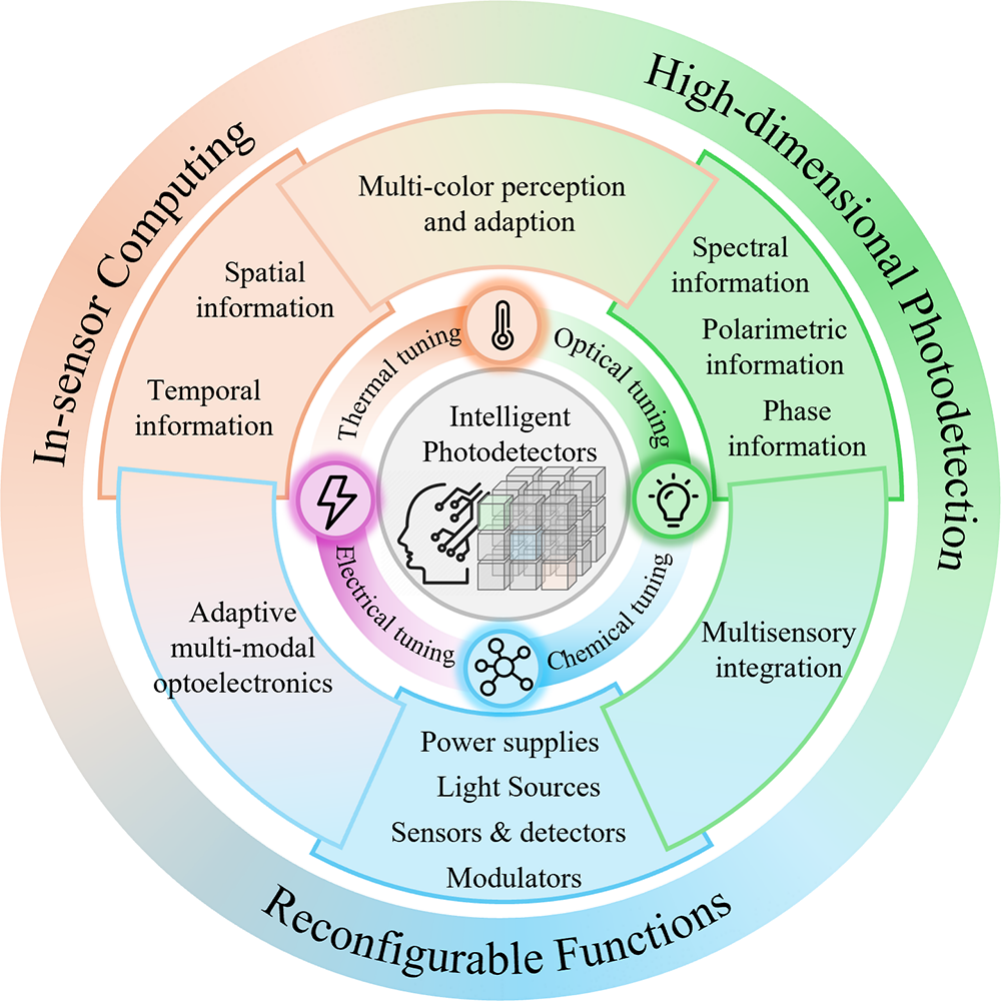
Directions for future intelligent photodetector development. Enhanced tunability across various responses and functional adaptability are paving the way for more advanced capabilities. Integrating multiple tunable variables presents a promising avenue for future innovations.

As the fields of materials science, optoelectronics, and computing continue to converge, we can expect the emergence of even more advanced and efficient intelligent photodetector systems. These innovations will be instrumental in meeting the evolving demands of modern technology, driving progress and opening new possibilities in a rapidly changing technological landscape.
